# In situ Synthesis of Supramolecular Polymers: Finding the Right Conditions when Combining Covalent and Non‐Covalent Synthesis

**DOI:** 10.1002/anie.202206729

**Published:** 2022-07-14

**Authors:** Tobias Schnitzer, S. A. H. Jansen, Mathijs F. J. Mabesoone, Ghislaine Vantomme, E. W. Meijer

**Affiliations:** ^1^ Institute of Complex Molecular Systems and Laboratory of Macromolecular and Organic Chemistry Eindhoven University of Technology P. O. Box 513 5600 MB Eindhoven The Netherlands

**Keywords:** Non-Covalent Synthesis, Supramolecular Polymers, Systems Chemistry

## Abstract

The combination of covalent and non‐covalent synthesis is omnipresent in nature and potentially enables access to new materials. Yet, the fundamental principles that govern such a synthesis are barely understood. Here, we demonstrate how even simple reaction mixtures behave surprisingly complex when covalent reactions are coupled to self‐assembly processes. Specifically, we study the reaction behavior of a system in which the in situ formation of discotic benzene‐1,3,5‐tricarboxamide (BTA) monomers is linked to an intertwined non‐covalent reaction network including self‐assembly into helical BTA polymers. This system shows an unexpected phase‐separation behavior in which an interplay of reactant/product concentrations, side‐products and solvent purity determines the system composition. We envision that these insights can bring us one step closer to how to design the synthesis of systems in a combined covalent/non‐covalent fashion.

## Graphical abstract

A combination of covalent and non‐covalent synthesis steps is promising to construct synthetic supramolecular systems. Yet, the fundamental principles that guide such an approach are barely understood. Here, the reaction behavior of in situ formed supramolecular polymers is studied, in which covalent reactions are intertwined with a non‐covalent reaction network leading to an unexpected phase‐separation behavior that determines the system composition.

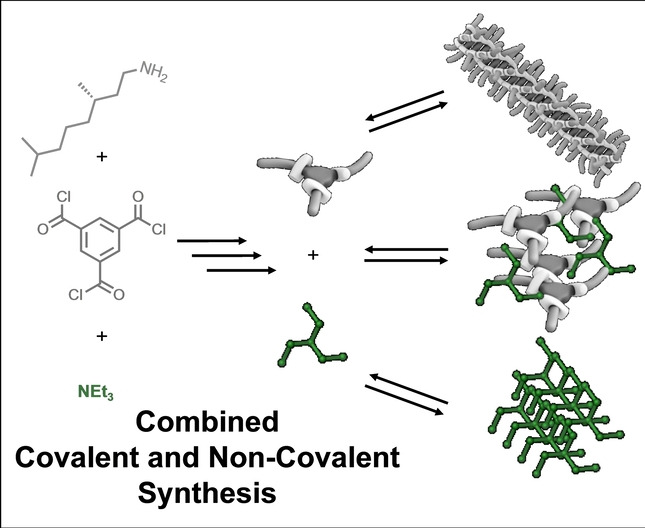

## Introduction

In recent years, the synthesis of complex molecular systems attracted increasing attention driven by our societal interest in e.g. efficient and sustainable solutions for energy storage, data transfer or understanding and mimicking life.[^1^, ^2^, ^3^, ^4^, ^5^, ^6^] The typical route to access such systems involves design, synthesis and purification of molecules, followed by their self‐assembly in a second, separate step. Thus, the structure of the molecular building blocks largely determines the complexity of the system.[^4^, ^5^, ^7^] This is in stark contrast to the generation of systems in nature, where—starting from often simple building blocks—a stepwise successive combination of covalent and non‐covalent synthetic steps leads to systems with emerging complexity.[^4^, ^5^] Transferring this bioinspired synthetic strategy to artificial systems is appealing, since it might provide access to novel structures and functions.[^4^, ^5^] This has been showcased in the field of systems chemistry, in which coupling of a cycle of covalent reactions to an assembly process creates systems with transient properties.[^3^, ^8^, ^9^, ^10^, ^11^, ^12^, ^13^, ^14^, ^15^, ^16^, ^17^, ^18^, ^19^, ^20^, ^21^] Yet, the number of examples also highlights the pitfalls that come along with a combined covalent/non‐covalent synthesis. The dynamicity of assemblies can amplify already subtle stimuli (e.g. temperature or solvent changes, additional reaction components) potentially stimulating drastic effects on the system.^[5]^ Especially constant changes of the reaction environment due to progressing conversion of starting materials to products might result in temporal effects on the assembly. Thus, fundamental mechanistic studies mapping out limitations and advantages of combined covalent/non‐covalent synthesis represent the first step towards a stepwise synthesis of complex molecular systems.

**Figure 1:**
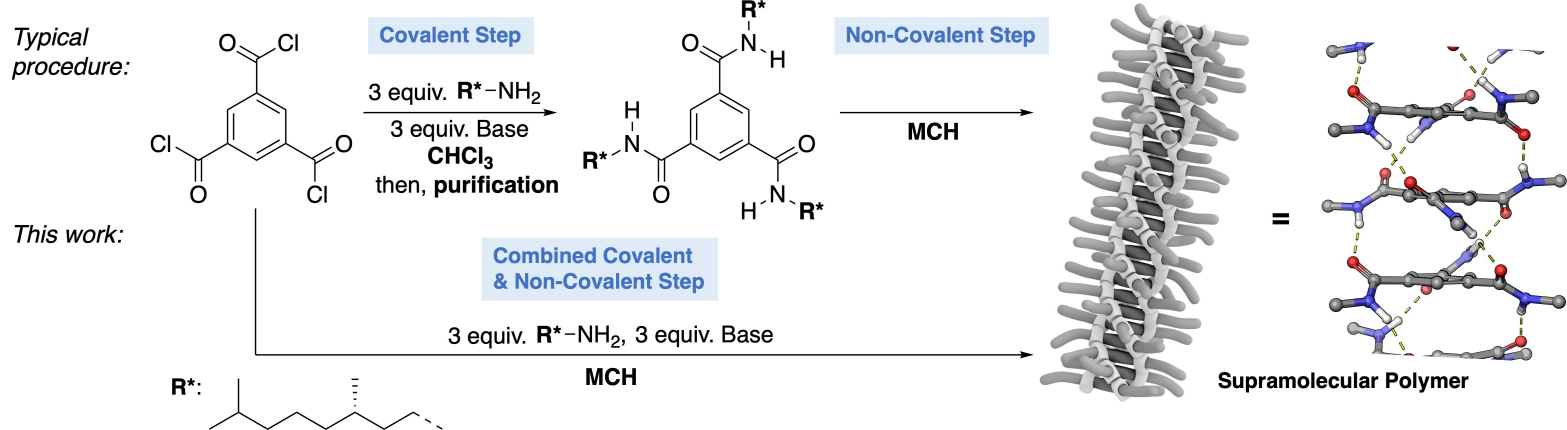
Typical procedure for the synthesis of benzene‐1,3,5‐tricarboxamide (BTA) monomers followed by purification and subsequent formation of the supramolecular polymers in a second, separate step. In this work we combine both steps, the covalent amidation and non‐covalent assembly and study the properties of the resulting multi‐component systems.

Benzene‐1,3,5‐tricarboxamides (BTAs) are *C_3_*‐symmetric, discotic molecules that form helical supramolecular polymers at μM and organogels at mM concentrations in alkanes.[^22^, ^23^, ^24^] The underlying assembly mechanism and conformational requirements of BTA building blocks have been studied in great detail.[^22^, ^23^, ^24^] The polymer formation is driven by intermolecular hydrogen bonds of the three amide groups and *π*‐stacking of the aromatic cores (Figure 1, right).[^22^, ^23^, ^24^] Chiral aliphatic side chains on the amide groups can bias the helical screw sense of the polymer, which enables monitoring of the polymer formation via e.g. circular dichroism (CD) spectroscopy.^[22]^ As BTA polymers adapt their most stable thermodynamic state in organic solvents within seconds at room temperature,^[24]^ they react quickly to stimuli and are ideal model systems to investigate the combination of covalent and non‐covalent synthesis steps.

Typically, BTA polymers are prepared in two separate steps (Figure 1): 1) covalent step—trifold amidation between trimesoic acid chloride and a primary amine in the presence of a base (in CHCl_3_ at mM concentrations of acid chloride, primary amine and base) and 2) non‐covalent steps—purification and assembling (in methylcyclohexane (MCH) at μM concentrations). Here, we combine both, the covalent and non‐covalent synthetic reaction step to study the formation of the supramolecular polymer system over time. We show that interplay of covalent and non‐covalent reaction kinetics, assembling of reaction products and phase separation processes lead to an unexpected system formation. Moreover, we highlight how already minute changes in the synthesis setup such as solvent purity or reagent solubility can be used to drive the system composition. Thus, our study provides a checklist of potential obstacles in a combined covalent/non‐covalent synthesis along with showcasing its potential to prepare novel systems.

## Results and Discussion

**Figure 2:**
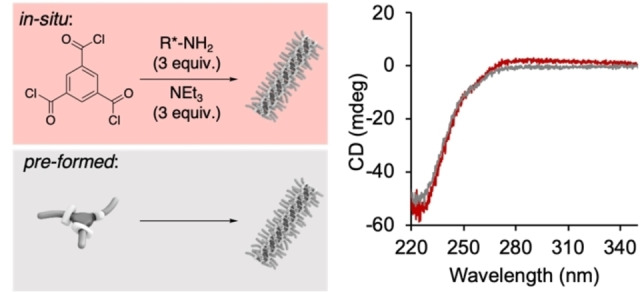
CD spectra indicative for the supramolecular polymers formed in the system of in situ formed BTA (red) and pre‐formed BTA (grey; 50 μM with respect to trimesoic acid chloride or pre‐formed BTA, respectively). Note, the trimesoic acid chloride starting material does not form supramolecular assemblies under these conditions (Figure S2).

We started our investigation on the amidation reaction of trimesoic acid chloride and enantiopure (*S*)‐dihydrocitronelyl amine using triethylamine as a base (Figure 1). To study the compatibility of the amidation reaction with the solvent MCH, a solution of the acid chloride was added to a solution of primary amine and base in this solvent (final concentrations: c(trimesoic acid chloride)=10 mM, c(primary amine)=30 mM, c(NEt_3_)=30 mM; for details see Supporting Information p. S4). Immediate gelation of the mixture and precipitation of the triethyl ammonium chloride salt suggested rapid conversion of the starting materials within seconds. This was supported by ^1^H nuclear magnetic resonance (NMR) spectroscopic analysis of the sample which showed only two spin systems corresponding to the in situ generated BTA and triethylammonium chloride (Figure S1). Encouraged by the high reaction rate of the amidation in MCH (final concertation of BTA=10 mM), we next performed the amidation reaction at 50 μM concentration. After 1 h, the reaction mixture showed a negative Cotton effect in the CD spectrum indicative for the formation of the supramolecular polymer. Comparison of the CD spectrum of a 50 μM solution of in situ‐formed BTA in MCH with that of a pure sample of pre‐formed (and purified) BTA as reference confirmed quantitative conversion to the tricarboxamide (Figure 2). This was also supported my mass spectrometric analysis of the reaction mixture, which showed no signals indicative for partial amidation and the formation of the mono‐ or di‐amide (see Supporting Information p. S7 and Figure S4).

**Figure 3:**
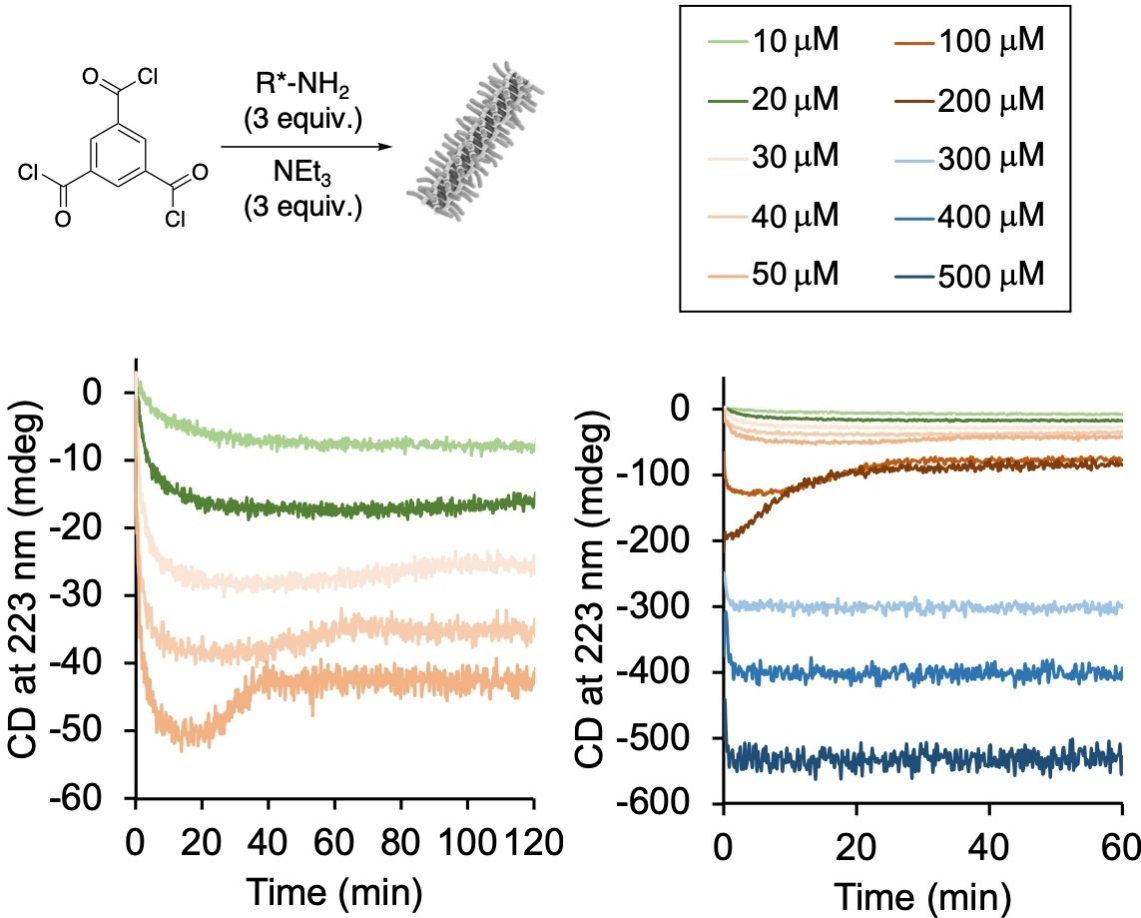
Kinetic analysis of the formation of supramolecular polymer by CD spectroscopy depending on the reactant concentration (concentrations refer to trimesoic acid chloride, water content of MCH ≈35 ppm).

To study the effect of the amidation reaction on the non‐covalent assembly, we followed the reaction progress via CD spectroscopy by monitoring the signal at 223 nm. Different reaction concentrations from 10–500 μM were used to investigate the effect of different reaction rates in the amidation reaction on the assembly (see Supporting Information 3.1). At low concentrations (10, 20 μM, with respect to trimesoic acid chloride), the obtained reaction profiles showed an exponential shape (quantitative conversion in ≈40 and ≈30 min), as expected for second order reactions (Figure 3). With increasing reaction concentrations (30–200 μM), the initial rates increase. However, the shapes of the kinetic traces are unexpected and remarkable: after the initial increase of the signal, a maximum of the negative Cotton effect was reached after <1–25 min, followed by decrease of the signal until a plateau is reached. The subsequent decrease of the signal is least pronounced at 30 μM, most pronounced at 200 μM reaction concentration. Most remarkably, when the concentration is further increased to 300–500 μM, the unexpected rate profile vanishes and the same exponential rate profile as for concentrations <30 μM is observed. This concentration dependency suggests an intermolecular reaction as the origin for the non‐classical reaction behavior observed in the in situ BTA synthesis.

**Figure 4:**
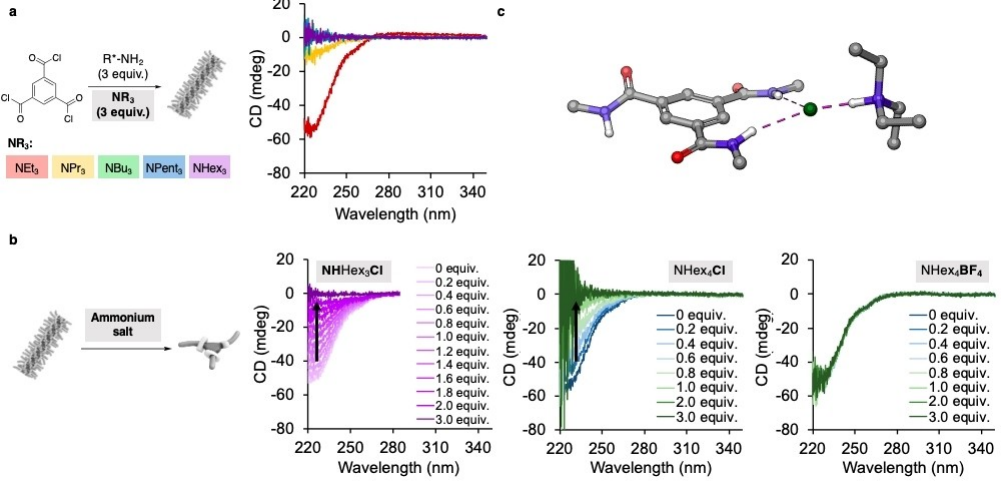
a) In situ BTA generation in the presence of different trialkyl amine bases monitored by CD spectroscopy after 1 h (MCH, 50 μM concentration of trimesoic acid chloride). b) Titration of trihexylammonium chloride (left), tetrahexylammonium chloride (middle) and tetrahexylammonium tetrafluoroborate (right) to a 50 μM solution of BTA in MCH. c) Computed lowest energy structure of a trimethyl benzene tricarboxamide and triethylammonium chloride (B3‐LYP‐D3, 6‐311G+*, vacuum). Microscopic analysis of the BTA/HNEt_3_Cl complex showed needle shaped non‐birefringent objects (Figure S11).

We reasoned that the surprising reaction profiles observed at different reactant concentrations (Figure 3) could originate either from interactions with the starting materials, the ammonium chloride side product or small amounts of water, which are known to be present in MCH. We first studied the effect of each reaction component on the assembly. CD spectroscopic titration experiments with up to 3 equivalents of either the acid chloride, the primary amine, or triethylamine starting materials (50 μM, MCH) indicated no interference with the assembly (Figure S5). Next, we focused on the ammonium chloride side product, which is formed in three equivalents over the course of the amidation reaction. As the low solubility of triethylammonium chloride prevents titration, we compared the reaction in the presence of NEt_3_ with that of NPr_3_, NBu_3_, NPent_3_, and NHex_3_, at a 50 μM concentration (for details see Supporting Information p. S7, for interaction of the polymer with the amine base, see Supporting Information Figure S6). We hypothesized that the variation of the alkyl length of the base will not affect the covalent reaction and thus assume quantitative conversion after 1 h analogous to NEt_3_. This was supported by mass spectrometric analysis of the reaction mixtures, which showed no evidence of unreacted acid chloride or formation of undesired mono‐/di‐amide side products (see Figure S4). Remarkably, only in the presence of NEt_3_ the expected Cotton effect was observed (Figure 4a), NPr_3_ gave the same signal with lower intensity, and NBu_3_, NPent_3_, and NHex_3_ showed no signal. This suggests that already 3 equivalents of ammonium salt are sufficient to fully depolymerize the BTA assembly. The differences in depolymerization strength of HNEt_3_Cl≪HNPr_3_Cl<HNBu_3_Cl≈HNPent_3_Cl≈HNHex_3_Cl were attributed to the solubility of the salts in MCH: while HNEt_3_Cl is almost insoluble, HNHex_3_Cl has a low but sufficient solubility to interact with the polymer. Indeed, HNHex_3_Cl was sufficiently soluble in MCH to perform a titration experiment, which showed full depletion of the CD signal upon addition of 3 equivalents HNHex_3_Cl (Figure 4b, left). Computational analysis of a series of titration experiments using a thermodynamic mass‐balance model for 1 : 1 binding suggested a binding energy of −33 kJ mol^−1^ (for details see Supporting Information 5.1).

To gain further insights into the interactions that cause the destabilization of the assembly, we performed computational analysis of the BTA*HNEt_3_Cl complex using a trimethyl amide model compound (see Supporting Information chapter 6). A conformational search (OPLS3 force field, vacuum) with subsequent geometry optimization using density functional theory (DFT; B3‐LYP‐D3, 6‐311G+*, vacuum) of the complex was performed with Schrödinger Macromodel and Jaguar, respectively.[^25^, ^26^, ^27^] The obtained optimized structure showed three hydrogen bonds which all involve the Cl^−^ anion: two are formed with the BTA amide NH‘s and one with the hydrogen of the ammonium cation (Figure 4c). Comparison of the energy gain by formation of the [(BTA)_*x*_(HNEt_3_Cl)_*y*_] complex versus that of a BTA dimer provided a ≈2.5 times higher energy for the latter (Figure S22).

Our computational findings suggest that the Cl^−^ anion is crucial for the interference with the BTA assembly.^[28]^ To gain experimental evidence for this hypothesis, we performed two additional titrations with NHex_4_Cl (Figure 4b, middle and right), of which only the Cl^−^ and not the NHex_4_^+^ can engage in hydrogen bonds, and NHex_4_BF_4_, in which the potentially coordinating anion is replaced by non‐coordinating BF_4_^−^. The titrations showed a decrease of the Cotton effect upon addition of NHex_4_Cl and no change in the presence of NHex_4_BF_4_. To further comprehend the interaction of the ammonium chloride with the BTA, we studied the interaction of NHex_4_Cl with the supramolecular polymer by IR (Figure S7) and ^1^H‐NMR spectroscopy (Figure S8). Both experiments show significant changes of the signals corresponding to the BTA amide moieties upon addition of the ammonium salt. Furthermore, we studied the precipitate formed in the in situ BTA formation by mass spectrometry (Figure S10). Reassuringly, this experiment revealed that the precipitate is composed of BTA and HNEt_3_Cl. All these experiments suggest that the Cl^−^ anion of HNEt_3_Cl is responsible for the depolymerization of the BTA assembly by the competitive sequestration of BTA monomers,^[29]^ while the ammonium cation serves as a solubilizer of the salt.

**Figure 5:**
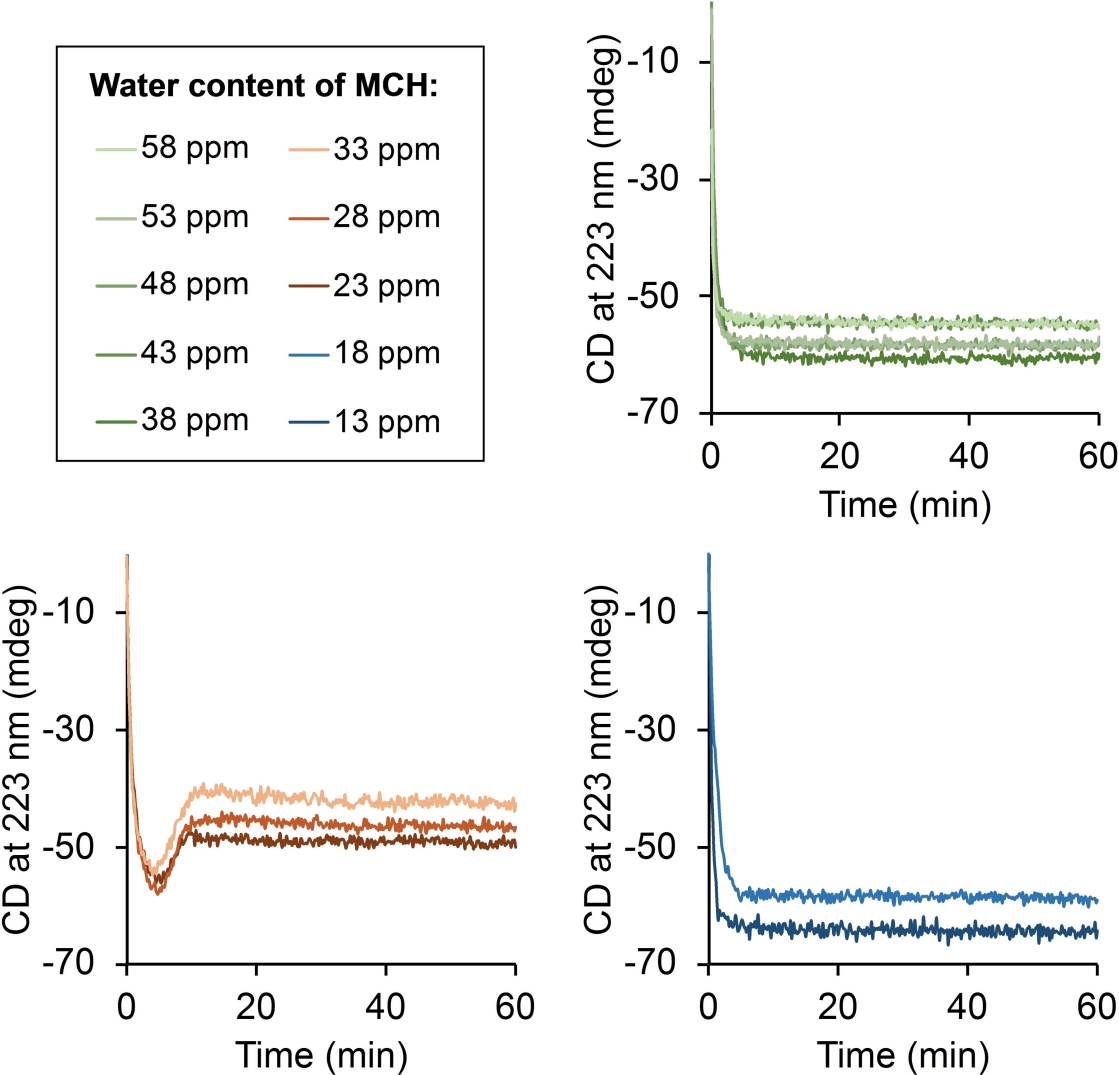
a) In situ BTA generation (50 μM trimesoic acid chloride, 3 equiv primary amine, 3 equiv NEt_3_) performed in MCH with varying water content.

Previous studies showed that minute amounts of water, present in MCH, can affect the assembling of discotic molecules. Yet, so far no interference with the polymerization of BTAs has been observed.^[30]^ We reasoned that in our system, the water content might be crucial for the assembly process, as the solubility of the ammonium salt is critical for the interference with the supramolecular polymer. In order to investigate this potential effect, we performed the in situ BTA formation (50 μM concentration) in MCH with varying water content (13–58 ppm, Figure 5, see Supporting Information 3.2). Remarkably, the observed reaction kinetic depends largely on the systems water content: in the presence of “high” amounts of water (38–58 ppm), the expected second‐order reaction kinetics is observed (similar to reactions at low concentrations). We attribute this to the well‐solubilized ammonium salt formed, which is engaged in H‐bonding with water instead of BTA molecules. Thus, no destabilization of the BTA over time is observed. From a critical water content of 23 ppm to 33 ppm the non‐classical reaction behavior based on interactions between the BTA and the ammonium salt is observed. While, at a low water content (13–18 ppm) again classical reaction behavior is observed presumably due to low solubility and precipitation of the ammonium salt. Thus, the phase separation which is critical for the system composition can be triggered not only due to changes in the concentration, but also the water content of the solvent.

**Figure 6:**
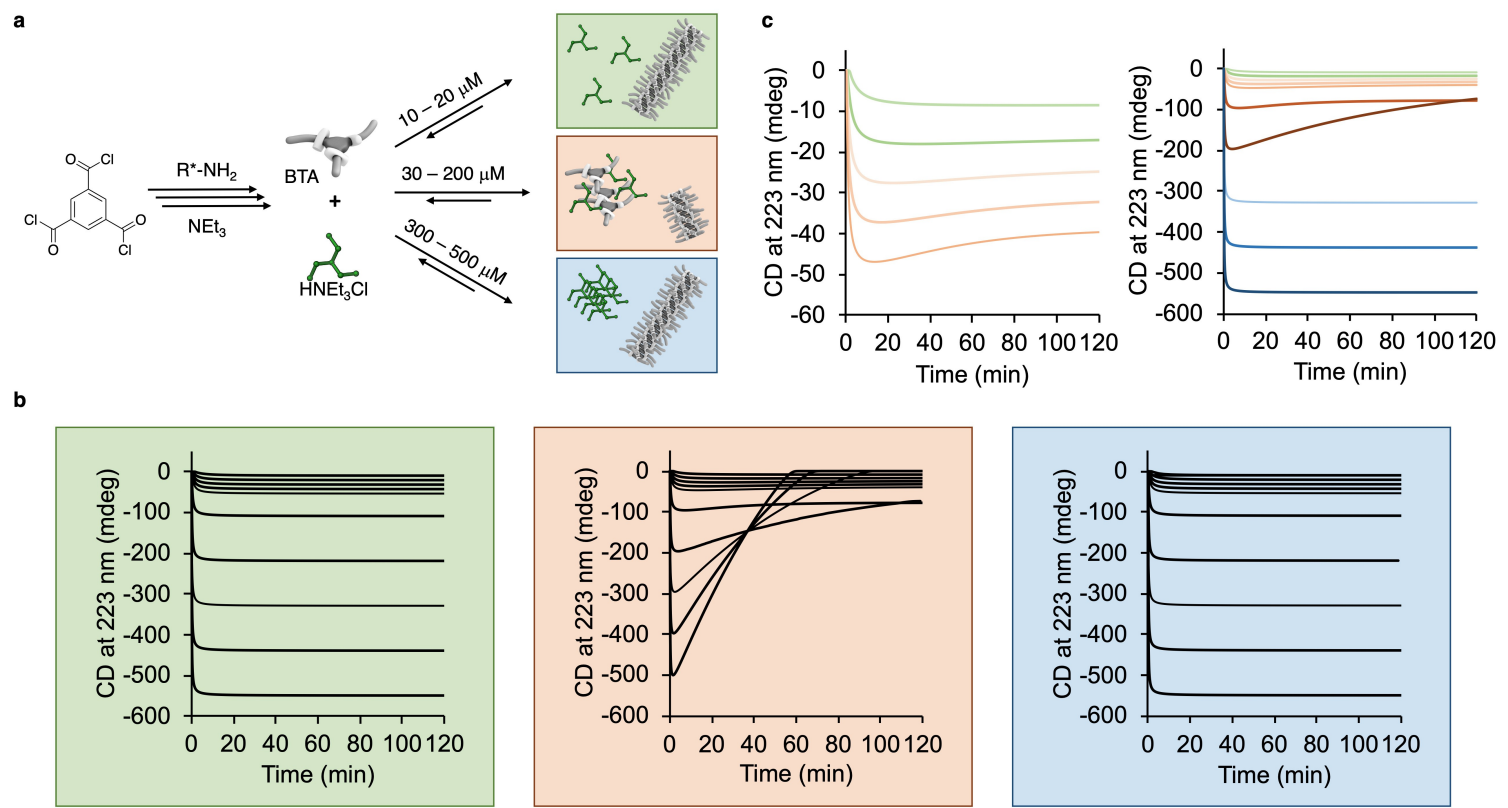
a) Proposed mechanism for the in situ generation of BTA‐based supramolecular polymers. b) Simulated reaction profiles considering that only one reaction pathway takes place: the BTA polymerization (left), the polymerization in presence of [(BTA)_x_(HNEt_3_Cl)_y_] complex formation (middle), the polymerization in presence of ammonium salt precipitation (right). c) Simulated reaction profiles at varying reactant concentrations (compare Figure 3 for color coding).

The combined DFT computational and experimental study of the system allowed us to identify four covalent/non‐covalent transformations: amidation reaction, BTA polymerization, HNEt_3_Cl precipitation, and [(BTA)_*x*_(HNEt_3_Cl)_*y*_] complex formation. With these reactions we can hypothesize the origin of the concentration‐dependent system composition over time by drawing the following mechanistic picture (Figure 6a): the amidation reaction yields BTA and three equivalents of HNEt_3_Cl within <1–20 min. At low concentrations (10, 20 μM), BTA and HNEt_3_Cl barely interact yielding unhindered, almost quantitative polymerization. When increasing the reaction concentration (30–200 μM), the interaction between BTA and HNEt_3_Cl becomes more pronounced. During the covalent reaction, a rapid increase of both products occurs until a critical concentration is reached. A sudden precipitation of the [(BTA)_*x*_(HNEt_3_Cl)_*y*_] complex then leads to a phase separation that causes partial depolymerization and decrease of the amount of assembly in solution. At high concentrations (300–500 μM), the amidation reaction reaches completion within <1 min providing high local concentration of the HNEt_3_Cl salt. As a consequence, rapid precipitation of the salt occurs preventing interaction with the BTA and leading to quantitative formation of the polymer.

To support our mechanistic hypotheses, we performed kinetic modeling of the covalent and non‐covalent reactions. We first separately modelled how each of the three non‐covalent reaction pathways influences the change of the CD signal over time at different concentrations (Figure 6b). When only the in situ formation of BTA and subsequent formation of supramolecular polymers is simulated, the expected reaction profiles indicate an almost quantitative polymerization for all concentrations (green box). Almost identical reaction profiles are obtained when the precipitation of the ammonium salt is considered (blue box). Only when the formation of the [(BTA)_*x*_(HNEt_3_Cl)_*y*_] complex is modelled, the sudden depletion of the CD signal is shown (orange box), which was also observed in the experimental reaction progress analysis. Remarkably, combining of the three non‐covalent reaction pathways by manual fitting (Figure 6c) allowed to replicate the concentration‐dependent trends of the experimental observations (see Supporting Information 5.2): At concentrations of 10 and 20 μM, the simulated CD signals follow a second‐order reaction profile. At concentrations of 30–200 μM, the simulated kinetic profile shows a similar trend as observed in the experiments, yet with a more gradual decrease of the absolute signal intensity. At high concentrations of 300–500 μM again exponential rate profiles are simulated without signal depletion, which is in agreement with the experimental curves. Overall, the simplified model used for the kinetic analysis supports our mechanistic hypotheses and highlights the phase separation of the supramolecular system components as key for the unexpected system composition over time.

## Conclusion

Our study on the in situ synthesis of BTA‐based supramolecular polymers showcased how covalent reactions steps intertwined with assembly processes and phase separations can lead to unexpected reaction behavior. Already subtle changes in the system design e.g. reactant concentration, reactant solubility or dryness of the solvent can have remarkable effects on the composition of the resulting system. Supramolecular chemistry advanced to a level at which the synthesis of complex systems can be driven by easily overlooked subtleties. It is therefore essential when applying new synthetic strategies to carefully perform systematic reaction parameter variation, identify the “sweet spot” for a certain reactivity window, and rigorously report the reaction conditions to reproducibly access synthetic systems. As a result, the use of well‐known approaches known for covalent synthesis (e.g. working in a glove box) might be recommended for future research in supramolecular chemistry. As a consequence, we suggest the term “non‐covalent synthesis” instead of self‐assembly or self‐aggregation for the work on complex multi‐component systems.^[4]^

## Author Contributions

T.S. and E.W.M. conceived and designed the overall project. T.S. conducted the experiments and DFT calculations. S.A.H.J. and M.F.J.M performed the reaction modelling. T.S. and E.W.M analyzed the data and prepared the manuscript with contributions from all authors.

## Conflict of interest

The authors declare no conflict of interest.

1

## Supporting information

As a service to our authors and readers, this journal provides supporting information supplied by the authors. Such materials are peer reviewed and may be re‐organized for online delivery, but are not copy‐edited or typeset. Technical support issues arising from supporting information (other than missing files) should be addressed to the authors.

Supporting Information

## Data Availability

The data that support the findings of this study are available in the supplementary material of this article.
