# Supplementary material for: In situ Synthesis of Supramolecular Polymers: Finding the Right Conditions when Combining Covalent and Non‐Covalent Synthesis
Source: Angew Chem Int Ed Engl. 2022 Jul 14;61(34):e202206729. doi: 10.1002/anie.202206729 (PMC9544088; doi:10.1002/anie.202206729)
Supplement: Supplementary file 1 — Supporting Information [file ANIE-61-0-s001.pdf]

Angewandte Chemie
Eine Zeitschrift der Gesellschaft Deutscher Chemiker

Supporting Information

In situ Synthesis of Supramolecular Polymers: Finding the Right Conditions when Combining Covalent and Non-Covalent Synthesis

*T. Schnitzer, S. A. H. Jansen, M. F. J. Mabesoone, G. Vantomme, E. W. Meijer**

Supporting Information

Contents

Supplementary Methods S3

1. General Aspects and Materials S3

2. NMR Spectroscopic Analysis of BTA Formation at mM Concentrations and Potential Assembly of Trimesoic Acid Chloride S4

3. Kinetic Studies on the *in-situ* BTA Formation S6

   3.1. Reaction Progress Analyses at Varying Reactant Concentrations S6

   3.2. Reaction Progress Analyses in MCH with Varying Water Content S6

   3.3. Reaction Progress Analyses in the Presence of Different Trialkylamine Bases S7

   3.4. Mass Spectrometric Analyses of the Reaction Mixtures in the Presence of Different Trialkylamine Bases S7

4. Study of the Interactions between Reaction Components/Analogs and the Supramolecular Polymer S9

   4.1. Studies in the Presence of Reactants S9

   4.2. Studies in the Presence of Different Ammonium Salts S10

      4.2.1. CD Spectroscopic Analyses in Solution S10

      4.2.2. IR Spectroscopic Analysis in Solution S11

      4.2.3. NMR Spectroscopic Analysis in Solution S12

      4.2.4. IR Spectroscopic Analysis of the Precipitate S13

      4.2.5. Mass Spectrometric Analysis of the Precipitate S14

      4.2.6. Microscopic Analysis of the Precipitate S14

5. Covalent and Non-covalent Reaction Modeling S15
5.1. Effect of Ammonium Salts on the Supramolecular Polymer S15
5.2. Kinetic Analysis of *in-situ* BTA Formation S19
6. DFT Computational Analysis S29
7. References S32

Supplementary Methods

1. General Aspects and Materials

Reagents and materials were of the highest commercially available grade and used without further purification. The dihydrocitronelyl amine and the BTA were prepared following the reported procedure.[1] ^{1}H-NMR spectra were recorded on a Varian Mercury Vx 400 MHz using deuterated chloroform purchased from Cambridge Isotope Laboratories. Chemical shifts (δ) are expressed in ppm and are referred to the signal of TMS (0.00 ppm). The water content of methylcyclohexane was determined *via* Karl Fischer titrations that were performed using a Mettler-Toledo C30 Coulometric KF Titrator loaded with CombiCoulomat Frit KF reagent (for cells with diaphragm, contains methanol, purchased from Merck). Approximately 1 g of sample was used for a typical single Karl Fischer titration. Ultraviolet and circular dichroism spectroscopy was performed using a Jasco J-815 spectrometer equipped with a Jasco PTC-348WI Peltier-type temperature controller. The sample holder was purged with nitrogen at a flow rate of 20 l min^{-1}. Quartz cuvettes (Hellma Analytics) with path lengths of 10 mm and 1 mm were used. MALDI-TOF spectra were recorded with Bruker Autoflex Speed using α-cyano-4-hydroxycinnamic acid (CHCA) or *trans*-2-[3- (4-*tert*-butylphenyl)-2-methyl-2-propenylidene]-malononitrile (DCBT) as matrix. FT-IR spectra were recorded on a PerkinElmer Spectrum One spectrometer Solution measurements were performed in a fluorine doped tin oxide coated glass slide cuvette (100 mm × 100 mm × 2.3 mm, surface resistivity 7 W/sq, 12 scans).

Sample Preparation: Stock solutions of the respective compounds were prepared by weighing 2 –10 mg of the compound and transferring it into a screw-capped vial followed by addition of the respective solvent using Gilson Microman Positive-Displacement Pipets. The samples were sonicated until homogeneous solutions were obtained.

2. NMR Spectroscopic Analysis of BTA Formation at mM Concentrations and Potential Assembly of Trimesoic Acid Chloride

Triethylamine (3 equiv., 0.3 mmol, 60 mM, 30.4 mg, 42 µL) and dihydrocitronelyl amine (3 equiv., 0.3 mmol, 60 mM, 47.2 mg) were dissolved in 5 mL MCH and stirred at room temperature. A solution of trimesoic acid chloride (1 equiv., 20 mM, 0.1 mmol, 26.5 mg) in MCH (5 mL) was added (final concentrations: acid chloride: 10 mM, triethylamine: 30 mM, dihydrocitronelyl amine: 30 mM). The solution was inverted 3-4 times (within 10 s) to properly mix the solution. Immediately after, a sample of 50 µL was taken and dissolved in $CDCl_3$ (750 µL) for ^1H NMR spectroscopic analysis. The NMR spectrum was recorded in a mixture of MCH/$CDCl_3$ 1:15. Signals of MCH appear in the region of 0.5-2.0 and do not hamper analysis of the signals indicative to determine the formation of the BTA (signals highlighted in yellow, orange, green) and ammonium salt product (signals highlighted in black). The absence of additional aromatic signals in the region of 8.0–8.5 indicate that neither acid chloride remained unreacted in the reaction mixture nor mono- or di-amide site-products were formed. Thus, we estimated – within the detection limit of the spectroscopic method – a conversion to the desired BTA of >98%. (Note: Signals corresponding to hydrogen atoms close to the aromatic core show lower intensity due to self-assembly into supramolecular polymers).

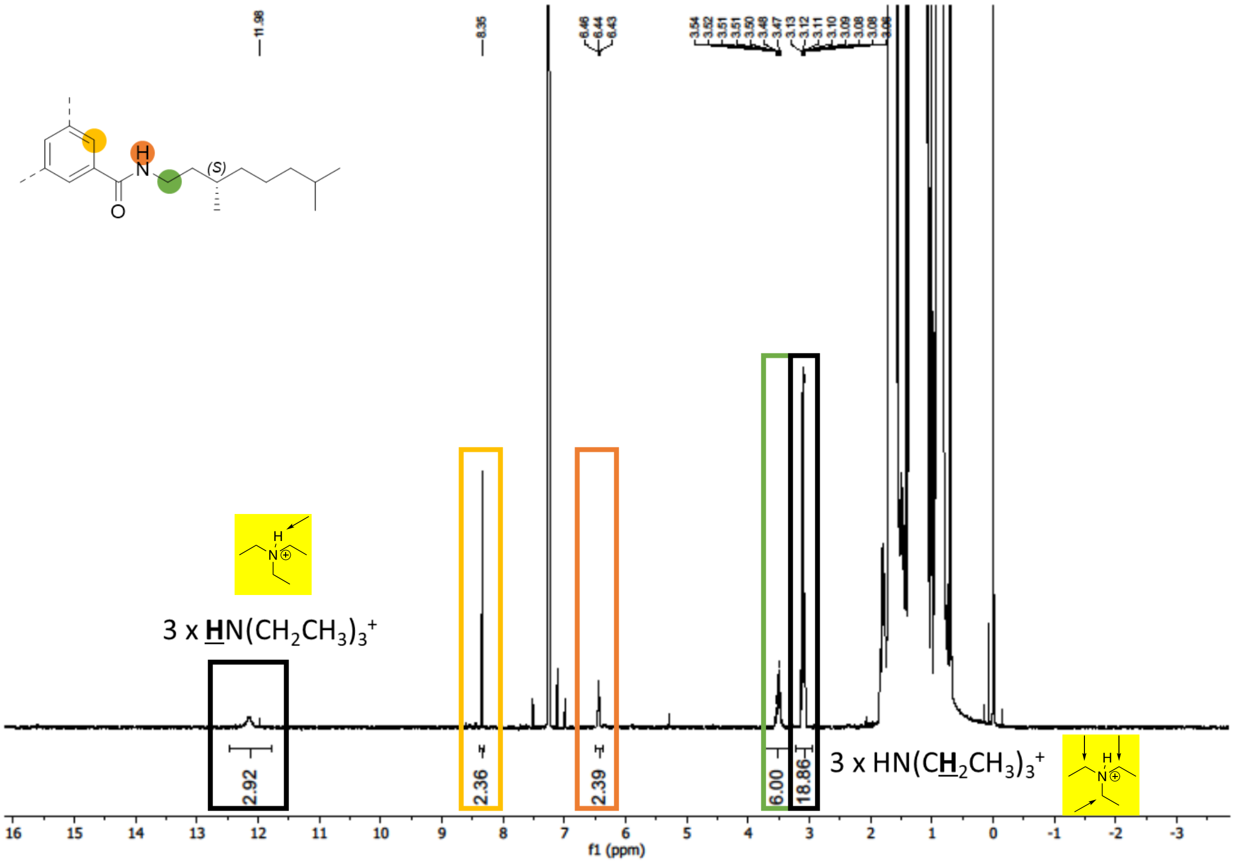

Figure S1: ^1H NMR spectrum of the crude reaction mixture of the *in-situ* BTA formation at mM concentrations. The spectrum was recorded at 300 MHz in $CDCl_3$/MCH 15:1.

Furthermore, we studied the potential assembly of trimesoic acid chloride (5 mM) in d[14]-MCH by ^{1}H NMR spectroscopy. At the same concentration, the signals of the BTA experience strong signal broadening which is indicative for self-assembly. In contrast, the acid chloride shows only one sharp signal corresponding to the aromatic CH`s of the benzene core. This suggests that neither at 5 mM concentration, nor at the concentration at which the in-situ BTA formation is studied (10-500 mM) self-assembly of the acid chloride.

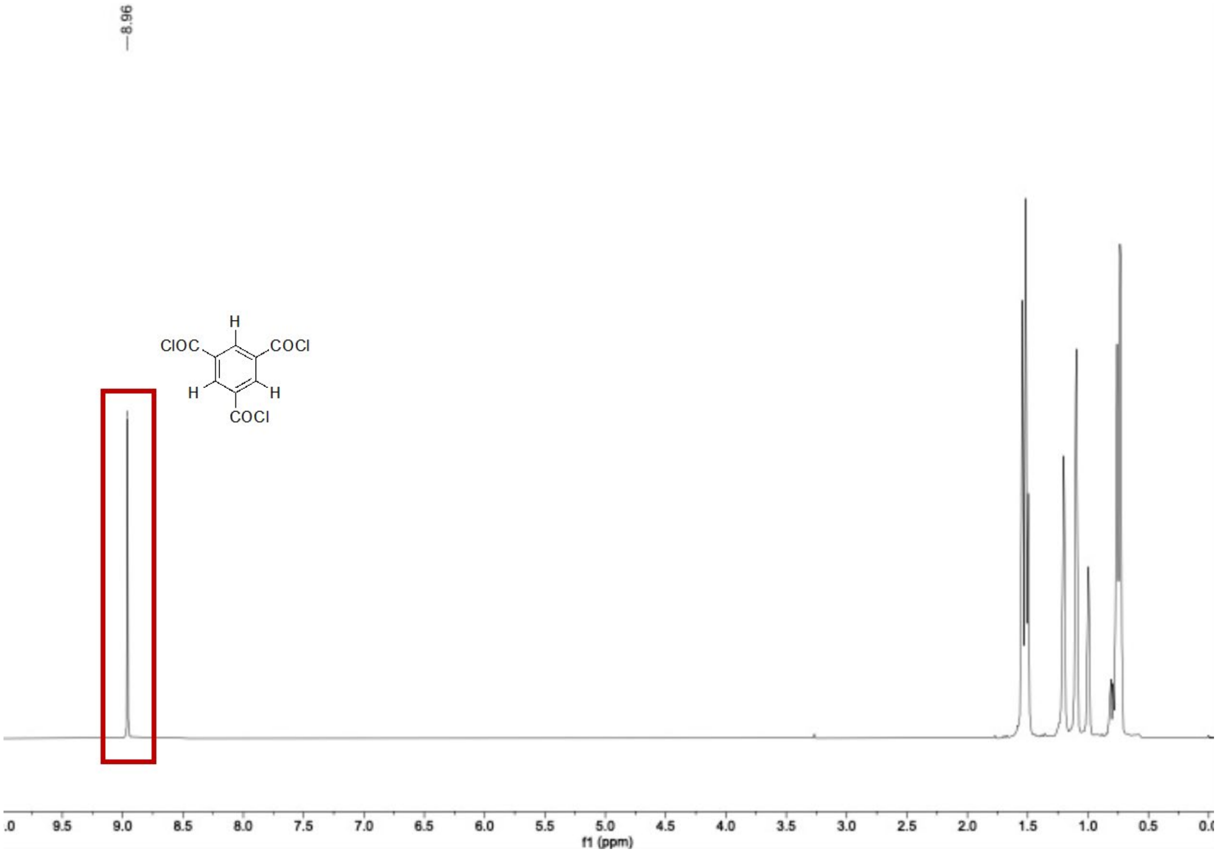

Figure S2: ^{1}H NMR spectrum of the crude reaction mixture of trimesoic acid chloride (5 mM) in d[14]-MCH recorded at 300 MHz. The signals between 0.5–2.0 correspond to residual non-deuterated MCH.

3. Kinetic Studies on the *in-situ* BTA Formation

3.1. Reaction Progress Analyses at Varying Reactant Concentrations

Reaction mixtures were prepared by adding the corresponding amount of triethylamine (3 equiv.) and dihydrocitronelyl amine (3 equiv.) from stock solutions to MCH. Inversion of the cuvette enabled well mixing of the components. Finally, the respective amount of trimesoic acid chloride (from stock solution; 1 equiv.) was added. The cuvette with the reaction mixture was inverted once to mix the components. Immediately after, the first data point for the kinetic measurement in the UV/CD spectrometer was taken. The reaction progress was monitored at 223 nm by UV and CD spectroscopy. The CD spectroscopic analysis is shown in the manuscript. Reactions at 10 – 50 μM were performed in a 10 mm cuvette and a total amount of 3 mL reaction solution, reactions at 100 – 500 μM were performed in a 1 mm cuvette and a total amount of 0.3 mL. Note, no "baseline" is recorded at 223 nm. Thus, the absorbance is given in arbitrary units. Furthermore, the UV absorbance at 223 nm is not only affected by the formation of the BTA (as it is in the case for the CD spectroscopic analysis), but also the consumption of triethylamine, dihydrocitronelyl amine and trimesoic acid chloride changes the absorbance.

It is crucial to mention, that already at these initial experiments, we realized that different reaction kinetics are obtained when different bottles of MCH are used. We later realized that this effect is based on the different amounts of water in MCH.

UV-spectroscopic analysis of the reactions at varying reactant concentrations:

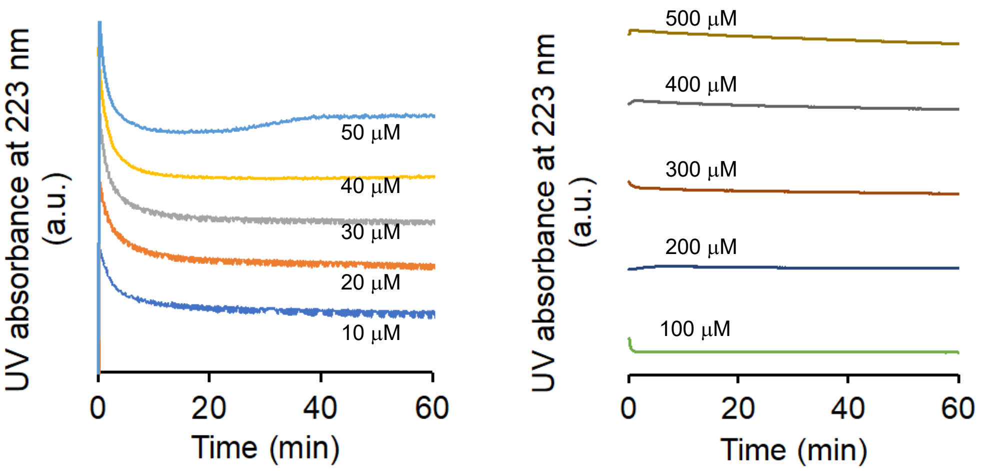

Figure S3: UV-vis spectroscopic monitoring of the reaction progress of the *in-situ* BTA formation at 10–500 μM (with respect to trimesoic acid chloride). The product formation was followed at 223 nm.

3.2. Reaction Progress Analyses in MCH with Varying Water Content

Reaction mixtures were prepared as described in 3.1. The amount of water in MCH was adjusted by mixing with water saturated MCH with dried MCH in different ratios. The amount of water was determined using Karl Fischer titration. The CD spectroscopic analysis of the different reactions is shown in the manuscript.

3.3. Reaction Progress Analyses in the Presence of Different Trialkylamine Bases

Reaction mixtures were prepared by adding the corresponding amount of trialkyl amine (3 equiv.) and dihydrocitronelyl amine (3 equiv.) from stock solutions to MCH. Inversion of the cuvette enables well mixing of the components. Finally, the respective amount of trimesoic acid chloride (from stock solution; 1 equiv.) was added. The cuvette with the reaction mixture was inverted once to mix the components. After 1 h, CD spectroscopic analysis of each reaction mixture (50 μM reaction mixture, 3 mL, 10 mm cuvette) was performed. The CD spectra of the different reactions are shown in the manuscript.

3.4. Mass Spectrometric Analyses of the Reaction Mixtures in the Presence of Different Trialkylamine Bases

Close to quantitative conversion of the *in-situ* BTA formation in the presence of different trialkylamine bases was confirmed by mass spectrometric analysis. Following the sample preparation in 3.3, reactions in the presence of NEt_3, NPr_3, NBu_3, $NPent_3$, and $NHex_3$ (concentration of trimesoic acid chloride: 50 μM; concentration of trialkylamine: 150 μM; concentration of dihydrocitronelyl amine: 150 μM) were prepared and studied by MALDI after 1 h. All reaction mixtures show a signal of m/z of 628.55 corresponding to the BTA ($[M+H]^+$; predicted $[M+H]^+$= 628.54 m/z). Furthermore, signals corresponding to the respective ammonium salts $HN\mathbf{R}_3^+$ are visible (**R=Et**: 102.07 m/z - predicted $[M+H]^+$= 102.13 m/z; **R=Pr**: 144.13 m/z - predicted $[M+H]^+$= 144.18 m/z; **R=Bu**: 186.20 m/z - predicted $[M+H]^+$= 186.22 m/z; **R=Pent**: 228.25 m/z - predicted $[M+H]^+$= 228.27 m/z; **R=Hex**: 270.30 m/z - predicted $[M+H]^+$= 270.32 m/z).

No signals corresponding to H^+/Na^+/K^+ adducts of dihydrocitronelyl amine (157.18 u), trimesoic acid chloride (263.91 u), the partially hydrolyzed mono-acid di-chloride (245.95 u) or di-acid mono-chloride (227.98 u) or trimesoic acid (210.02 u) were observed. Also, no indications for partial amidation, hence the mono-amide (bearing two acid chlorides moieties: 385.12 u; bearing one acid chloride and one carboxylic acid moieties: 367.16 u; bearing two carboxylic acid moieties: 349.19 u) or di-amide (bearing one acid chloride moieties: 506.33 u; bearing one carboxylic acid moieties: 488.36 u) were observed. *Note, the masses for [M] (not adducts) are listed for the compounds, which were not observed by MALDI.*

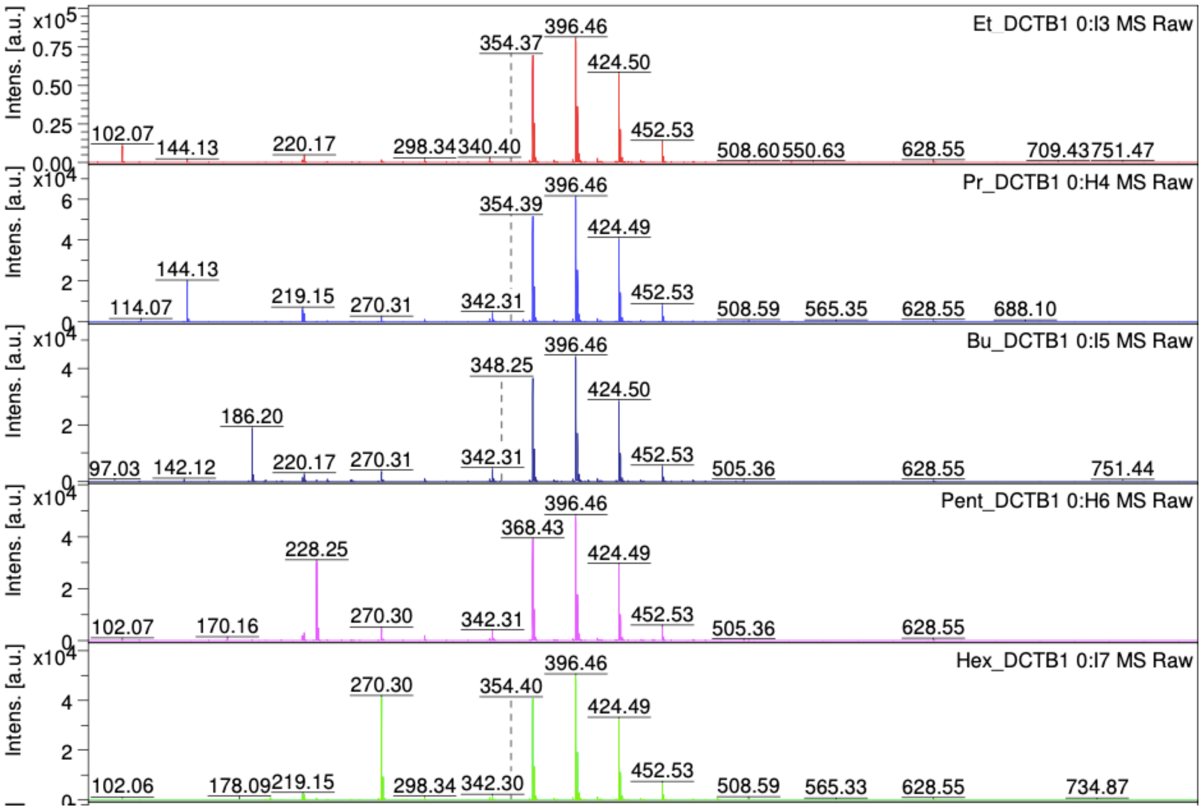

Figure S4: Mass spectra of the crude reaction mixtures of the *in-situ* BTA formation in the presence of different trialkyl amine bases (reaction concentrations of 50 μM with respect to trimesoic acid chloride). The measurement was recorded after 1 h reaction time. Note, ammonium ions show much higher signal intensity than BTA, which does now allow to quantify product formation.

4. Study of the Interactions between Reaction Components/Analogs and the Supramolecular Polymer

4.1. Studies in the Presence of Reactants

A 50 µM solution of BTA in MCH (~35 ppm water, total volume: 3 mL) was prepared in a 10 mm cuvette from the corresponding stock solution. The titration of triethyl amine, dihydrocitronelyl amine (R*-NH_2) and trimesoic acid chloride was performed by adding respective amounts of stock solutions (approx.1–2 µL for 0.2 equiv.). Inversion of the cuvette after each addition step provides homogeneous solutions. CD Spectra were immediately taken at room temperature. Note: addition of 2 and 3 equiv. of trimesoic acid chloride leads to detector saturation. Thus, the corresponding spectra are only shown for wavelengths >250 nm. The titration experiments indicate no interreference of the reactants with the supramolecular polymer.

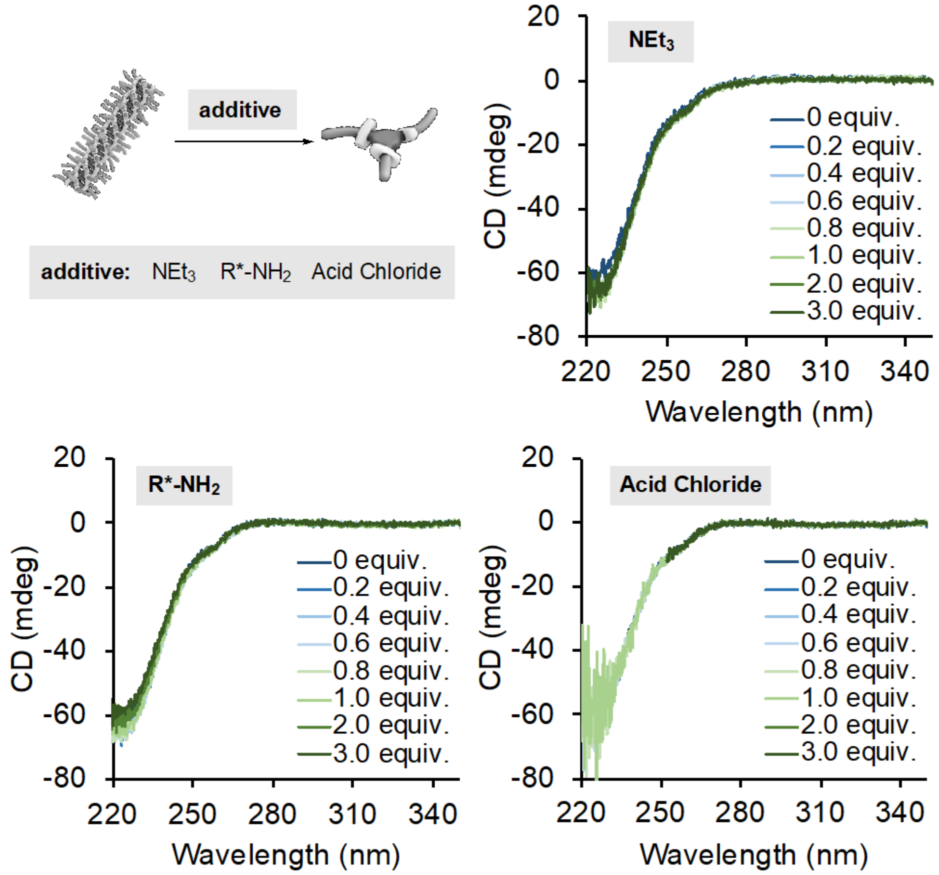

Figure S5: CD spectra of BTA (50 µM) in MCH in the presence of different equivalents of reactants.

Furthermore, we studied the effect of trialkyl amine bases with different alkyl chain lengths on the supramolecular polymer. For this purpose, CD spectra of a 50 µM solution of BTA in MCH (10 mm cuvette) in the presence of 3 equivalents (hence 150 µM) of NPr_3, NBu_3, $NPent_3$, and $NHex_3$ were recorded. No changes of the spectra upon addition of base indicate that no base-induced disruption of the supramolecular polymer occurs.

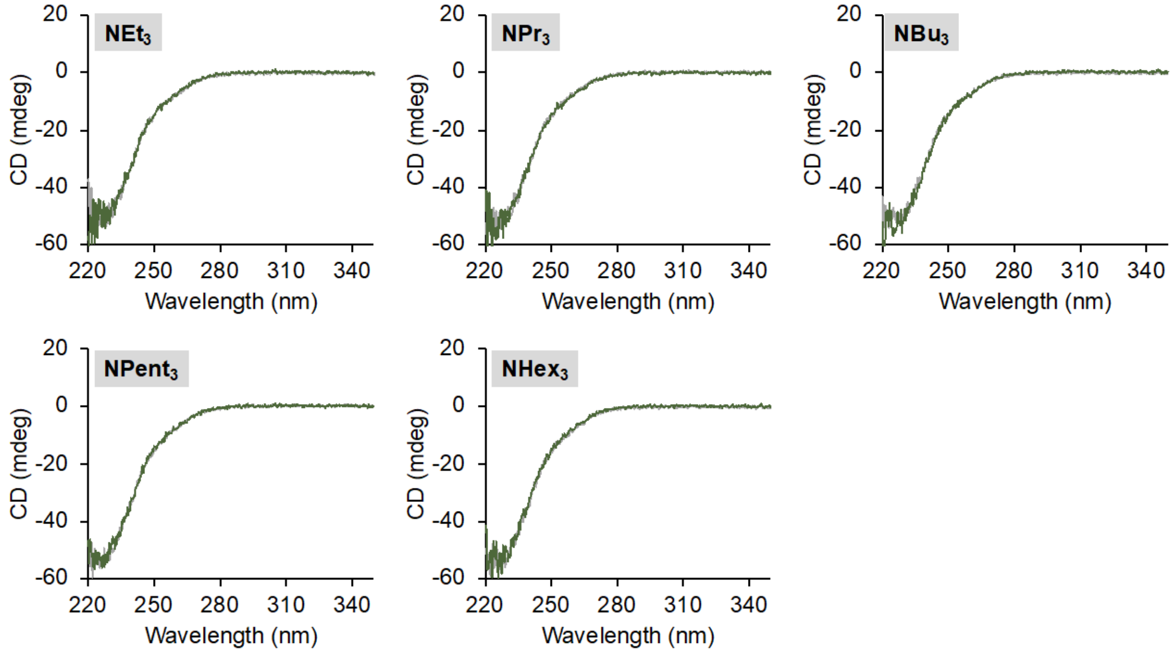

Figure S6: CD spectra of BTA (50 µM) in MCH in the absence (grey) and presence (green) of 3.0 equiv. of the respective trialkyl amines.

4.2. Studies in the Presence of Different Ammonium Salts

4.2.1. CD Spectroscopic Analyses in Solution

A 50 µM solution of BTA in MCH (~35 ppm water, total volume: 3 mL) was prepared in a 10 mm cuvette from the corresponding stock solution. The titration of trihexylammonium chloride, tetrahexylammonium chloride and tetrahexylammonium tetrafluoroborate was performed by adding respective amounts of stock solutions (approx. 1–2 µL for 0.2 equiv.). Inversion of the cuvette after each addition step provides homogeneous solutions. CD spectra were immediately taken at room temperature. The CD spectroscopic analysis of the titrations is shown in the manuscript.

4.2.2. IR Spectroscopic Analysis in Solution

To support binding between the BTA and the chloride anion of the formed ammonium salt, IR spectra of a 5 mM solution of BTA in MCH was recorded in the presence and absence of $NHex_4Cl$ (3 equiv., 15 mM). Upon addition of the ammonium salt, the vibrations of the amide moieties of the BTA shift, supporting that the chloride anion in interacts with the amide groups of the BTA.

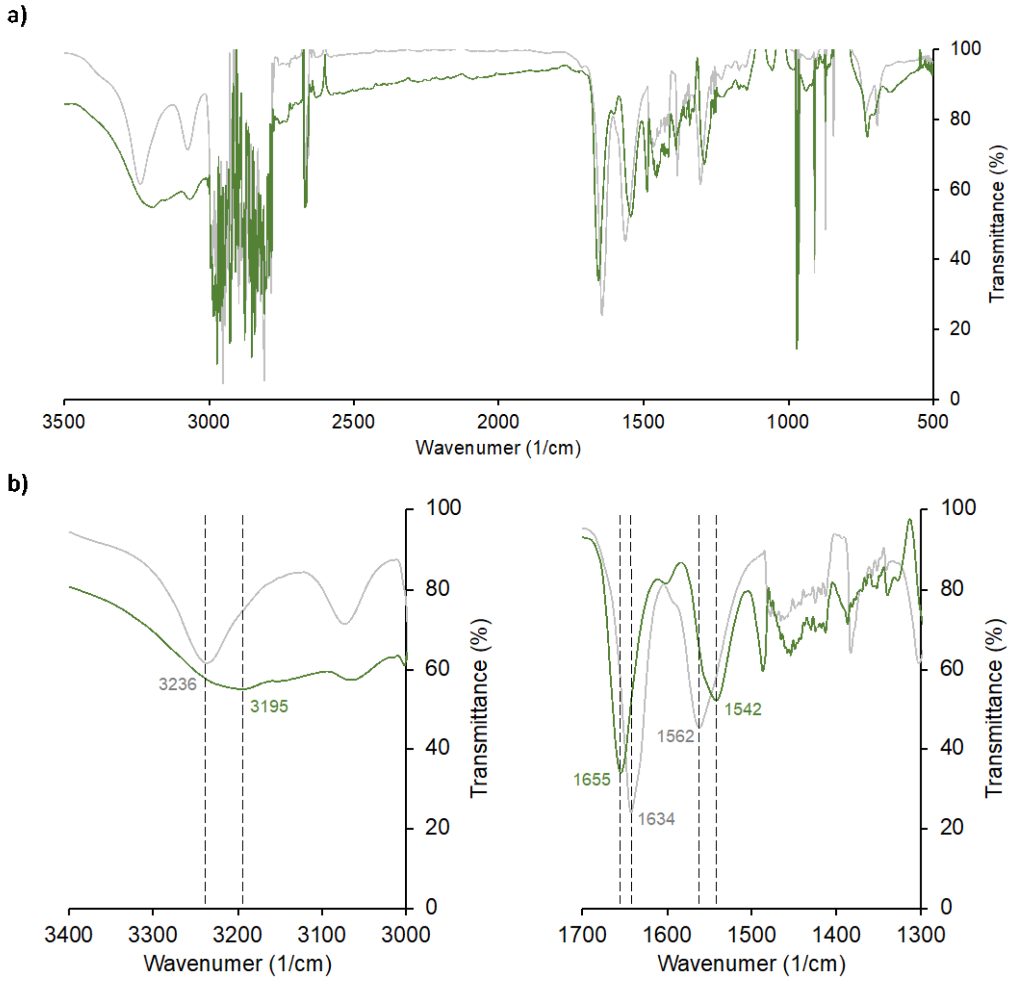

Figure S7: IR spectra of BTA (5 mM) in MCH in the absence and presence of $NHex_4Cl$ (3 equiv., 15 mM).

4.2.3. NMR Spectroscopic Analysis in Solution

To support binding between the BTA and the chloride anion of the formed ammonium salt, ^{1}H NMR spectra of a 5 mM solution of BTA in d^{14}-MCH was recorded in the presence and absence of NHex$_4$Cl (3 equiv., 15 mM). The spectrum of the pure BTA shows two broad signals at ~7.55 ppm and ~8.15 ppm corresponding to the amide NH`s (highlighted in orange) and the aromatic CH`s (highlighted in green) of the BTA. The broad signals indicate the formation of a supramolecular assembly. Upon addition of NHex$_4$Cl, the signal of the amide NH`s at ~7.55 ppm disappear (or decrease in intensity), which indicates a change of the chemical environment of the amide NH`s in the BTA and interaction with the chloride anion of NHex$_4$Cl.

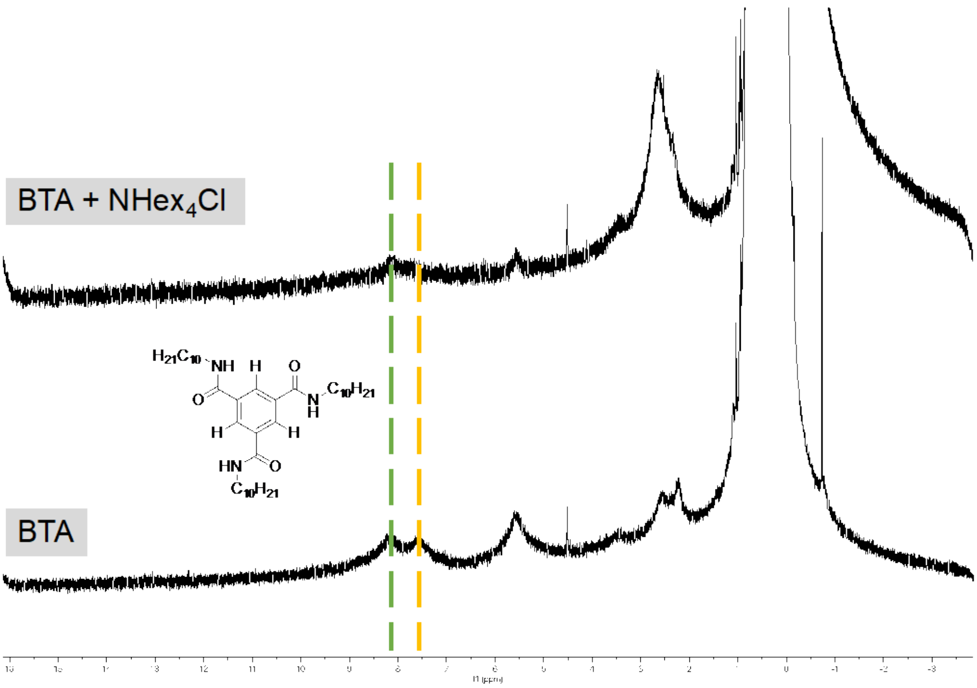

Figure S8: ^{1}H NMR spectra recorded at 300 MHz of BTA (5 mM) in d^{14}-MCH in the absence (bottom) and presence (top) of NHex$_4$Cl (3 equiv., 15 mM).

4.2.4. IR Spectroscopic Analysis of the Precipitate

The BTA/$HNEt_3Cl$ precipitate from a 50 μM *in-situ* BTA formation was studied by IR spectroscopy. For this purpose, 30 mL reaction volume was centrifuged (4000 rpm, 5 min). After removal of the supernatant, the precipitate was dried at rt overnight (at ambient atmosphere) and studied by IR spectroscopy. Comparison of the IR spectra of pure BTA with that of the precipitate showed only minute differences in the signal minima at 3224 cm^{-1} (BTA) versus 3223 cm^{-1} (precipitate) for the amide ν(N-H) vibration. While this measurement clearly shows that the BTA is part of the precipitate, the low concentration of BTA in the precipitate do not suffice to identify the exact binding mode in the precipitate. Furthermore, drying of the precipitate might also lead to disassembly of the formed co-assembly. However, analysis of the precipitate in solution was not successful.

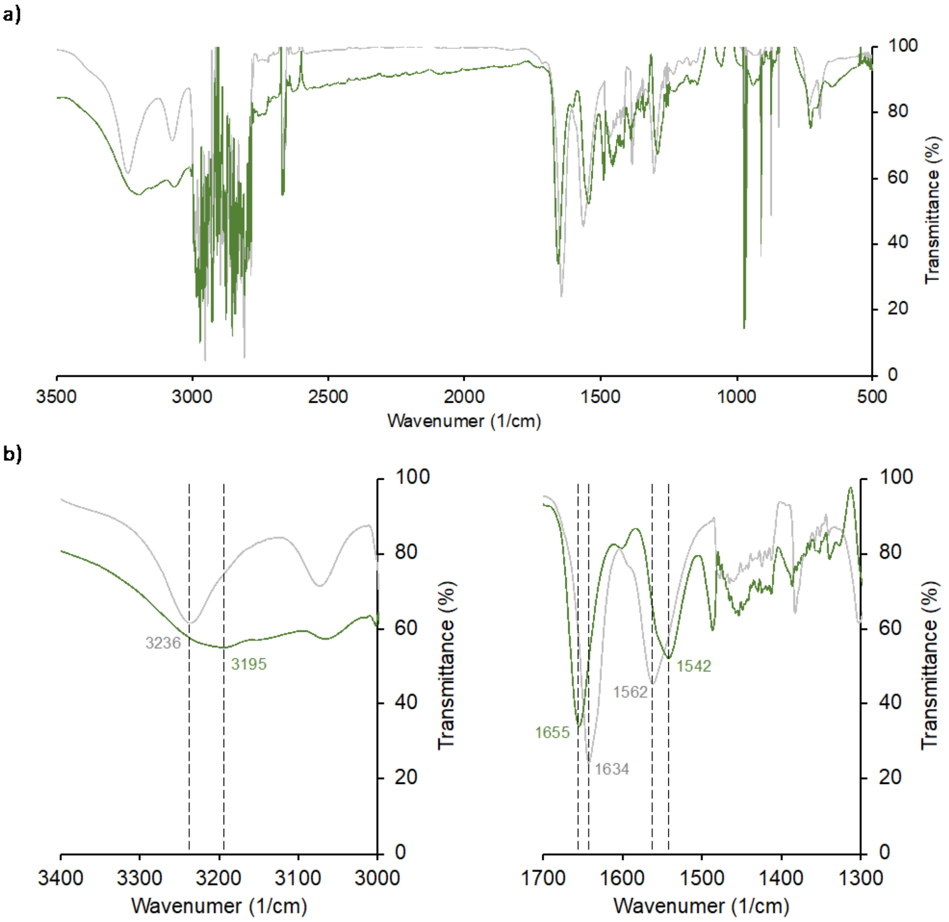

Figure S9: Comparison of the IR spectra of pure BTA and the precipitate obtained from the *in-situ* BTA formation at 50 μM concentration.

4.2.5. Mass Spectrometric Analysis of the Precipitate

The same precipitate that was studied in 4.2.4 was also analyzed by mass spectrometry. As expected, the signal of the $HNEt_3^+$ ($[M+H]^+$= 102.07 m/z - predicted $[M+H]^+$= 102.13 m/z) is clearly visible. The signal of the BTA is also visible but significantly smaller ($[M+H]^+$= 628.08 m/z - predicted $[M+H]^+$= 628.54 m/z). We attributed this to two factors: a) the lower visibility of the ammonium ion versus the BTA-adduct (compare 3.4), b) the reaction analysis by CD spectroscopy of the 50 μM *in-situ* BTA formation suggests that about 10-20% of the total BTA concentration is present in the precipitate. Thus, the precipitate contains a large excess of $HNEt_3Cl$.

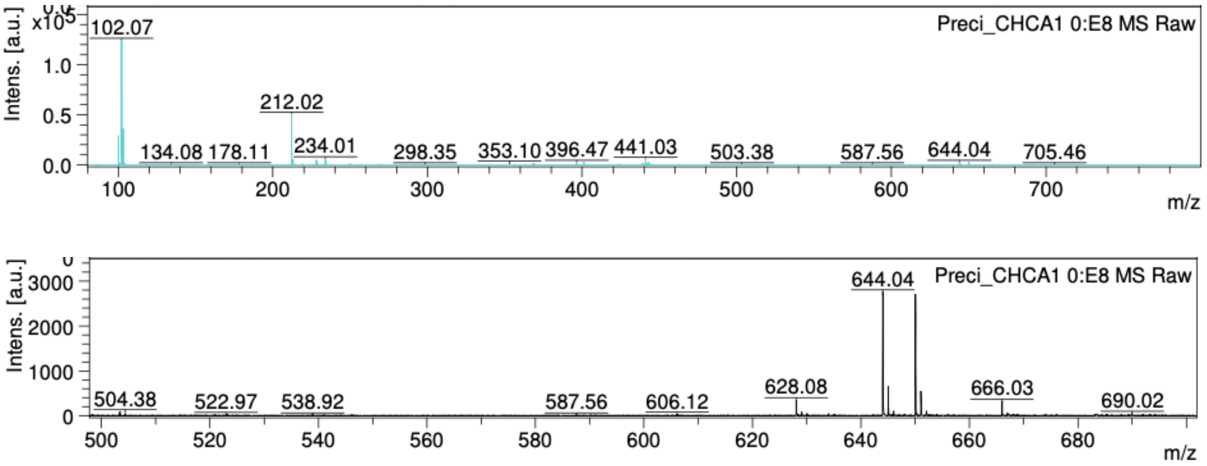

Figure S10: Mass spectrometric analysis of the precipitate obtained from the *in-situ* BTA formation at 50 μM concentration.

4.2.6. Microscopic Analysis of the Precipitate

The BTA/$HNEt_3Cl$ precipitate from a 50 μM *in-situ* BTA formation (compare 4.2.4) was investigated by microscopy before centrifugation and drying. The precipitate appears as needle shaped objects (length approx. 500 μm) which are not birefringent.

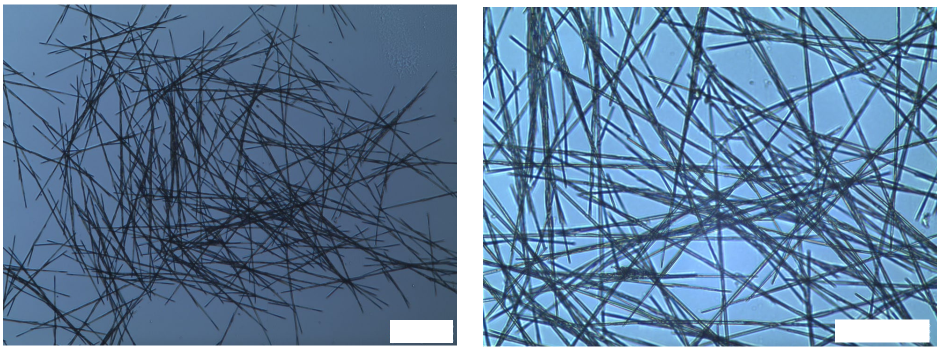

Figure S11: Microscopic analysis of the precipitate obtained from the *in-situ* BTA formation at 50 μM concentration (left: magnification 16 x 0.8; right: magnification 25 x 1.25, scale bars equal 100 μm).

5. Covalent and Non-covalent Reaction Modeling

5.1. Effect of Ammonium Salts on the Supramolecular Polymer

The complexation of BTA with $NHHex_3Cl$ and $NHex_4Cl$ are modelled using a mass-balance model in which the polymerization of the BTAs is in thermodynamic equilibrium with a competing complexation of the BTA with the ammonium chloride to form a BTA/ammonium salt complex. This complex does not take part in the polymerization. The polymerization is described by the following equilibria:

$$\begin{aligned}
&M_1 + M_1 \underset{}{\overset{K_{n,M}}{\rightleftharpoons}} M_2 \\
&M_2 + M_1 \overset{K_{e,M}}{\rightleftharpoons} M_3 \\
&M_3 + M_1 \overset{K_{e,M}}{\rightleftharpoons} M_4 \\
&\quad\vdots \\
&M_i + M_1 \overset{K_{e,M}}{\rightleftharpoons} M_{i+1} \\
&\quad\vdots
\end{aligned} \tag{1}$$

and the complexation to the ammonium salt is described by the following equilibrium:

$$M_1 + T \overset{K_{dim}}{\rightleftharpoons} M_1 \cdot T \tag{2}$$

with $K_{n,\,M}$ and $K_{e,\,M}$ the equilibrium constants for nucleation and elongation of the BTA polymerization, respectively, and K_{dim} the equilibrium constant for the complexation between the BTA monomer, M_1, and the ammonium salt, T.

The total concentration of BTA molecules in the system can then be expressed by:[2]

$$\begin{aligned}
[M]_{tot} &= [M]_{pol} + [M]_{complex} = \sum_{n=1}^{\infty} i\,[M_i] + [M_1 \cdot T] \\
&= \frac{\sigma[M_1]}{(1 - K_{e,\,M}[M_1])^2} - \sigma[M_1] + K_{dim}[M_1][T]
\end{aligned} \tag{3}$$

with $[M]_{pol}$ and $[M]_{complex}$ the concentration of BTA monomers in the polymers and $M_1 \cdot T$ complex, respectively, and σ the cooperativity parameter $\sigma = K_{n,\,M}/K_{e,\,M}$. The total concentration of the ammonium salt is given by:

$$[T]_{tot} = [T] + [T]_{complex} = [T] + K_{dim}[M_1 \cdot T] \tag{4}$$

with [T] the concentration of free ammonium salt. Eqs. (3) and (4) are solved using a nested binary search algorithm[3,4] in Matlab R2019a.

The model is fitted to all experimental data between 10 and 40 μM BTA simultaneously using Matlab's lsqnonlin function with the Levenberg-Marquardt algorithm. The fit is performed for 200 initial guesses for K_{dim} obtained from randomly sampling the Gibbs free energy of the complexation between −20 and −40 kJ/mol. The vector for which the norm of the residual vector, which contains the difference between every measured and calculated datapoint, is minimal is selected as best fit.

The Gibbs free energy and nucleation penalty, NP=exp(σ/(RT)) with R and T the gas constant and temperature, respectively, of the BTA polymerization are obtained from de Windt et al.[5] and set at −33.4 and 3.4 kJ/mol, respectively.

Results from the fits of the titration experiments

From the fits of the CD intensity at 222 nm to the thermodynamic mass-balance model described above, the Gibbs free energy of interaction of the 1:1 binding of M_1 with T is determined at −32.96±0.06 kJ/mol for $HNHex_3Cl$ as amine and at –30.23±0.06 kJ/mol for $NHex_4Cl$ as amine.

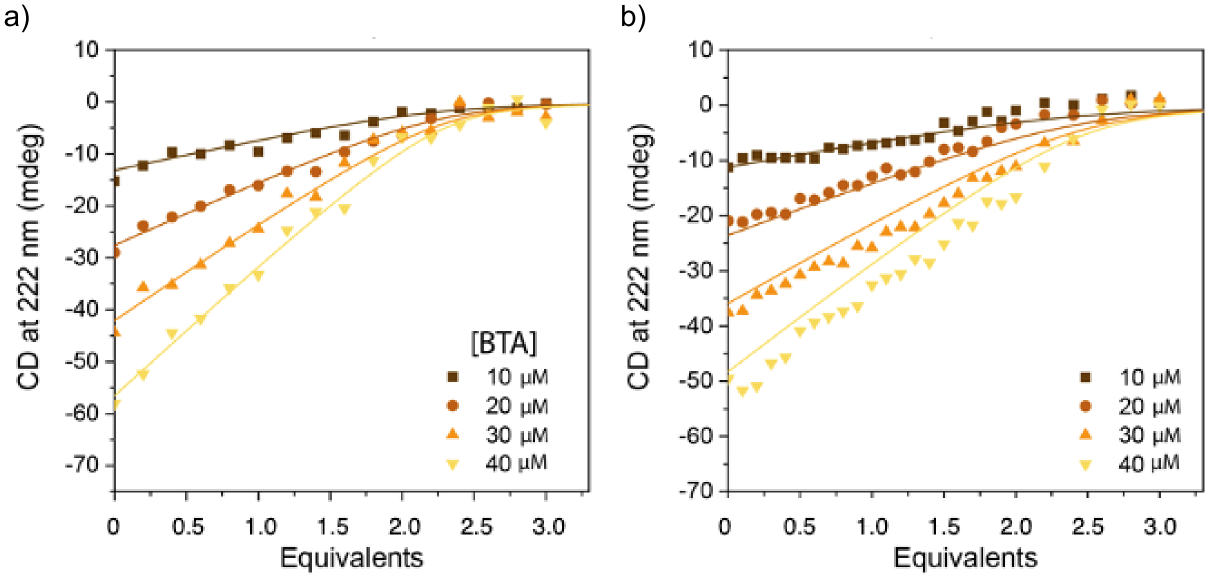

Figure S12: Fits of the CD intensity at 222 nm at 0–3.0 equivalents of $HNHex_3Cl$ (a) and Hex_4NCl (b) in MCH with 35 ppm H_2O. The symbols indicate the experimental data and the lines indicate the fits to the thermodynamic mass-balance model.

Spectra of the titration experiments:

Titration experiments were performed as described in 4.2.1.

HNHex$_3$Cl

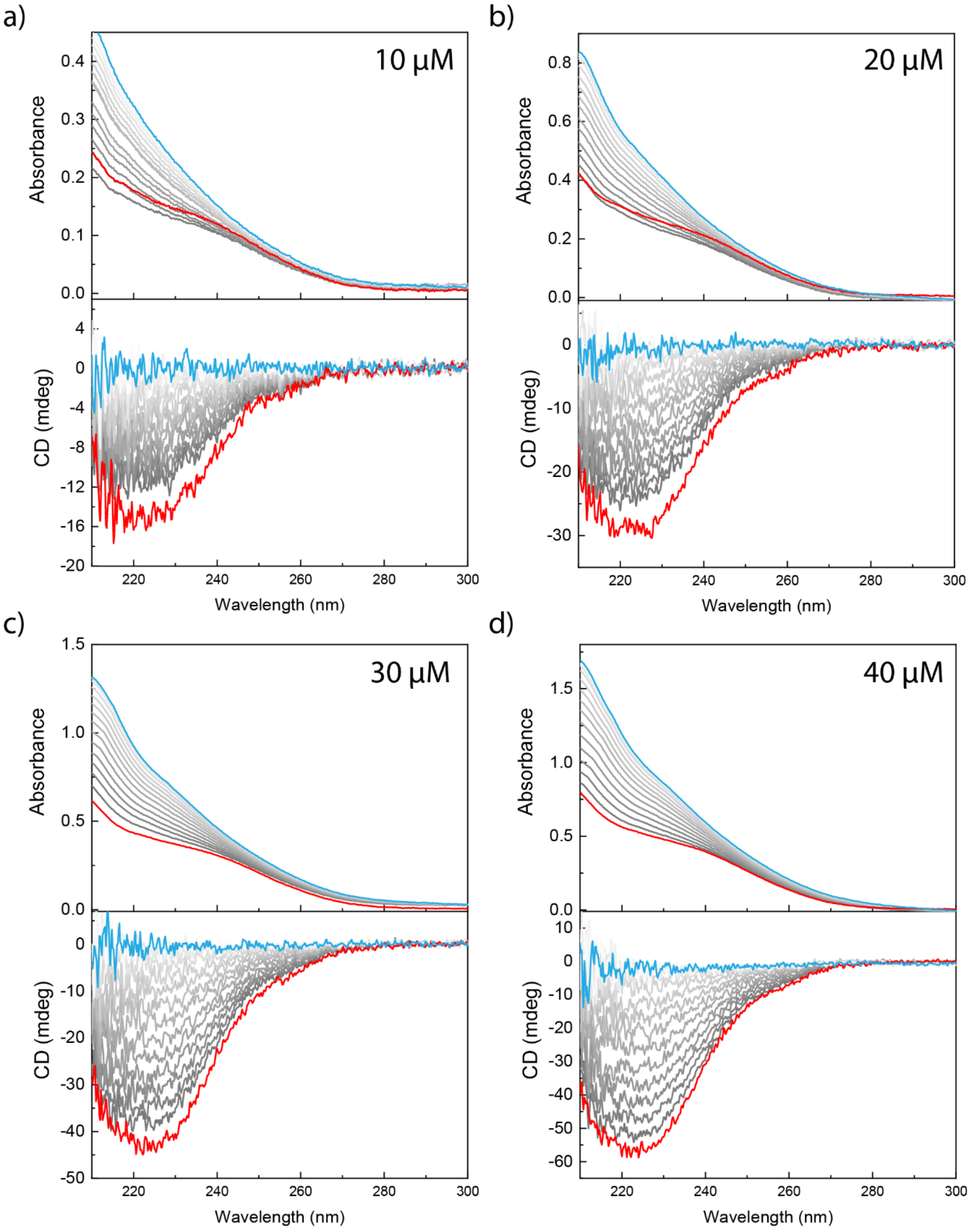

Figure S13: UV (top panels) and CD (bottom panels) spectra of the samples in the titration of BTA with HNHex$_3$Cl at 10 (a), 20 (b), 30 (c) and 40 (d) µM BTA. The red spectrum indicates the sample containing 0 equivalents HNHex$_3$Cl and the blue spectrum indicates the spectrum with 3.0 equivalents of the ammonium salt, with the increasing grayscale indicating the increasing intermediate equivalents.

$NHex_4Cl$

Titration experiments were performed as described in 4.2.1.

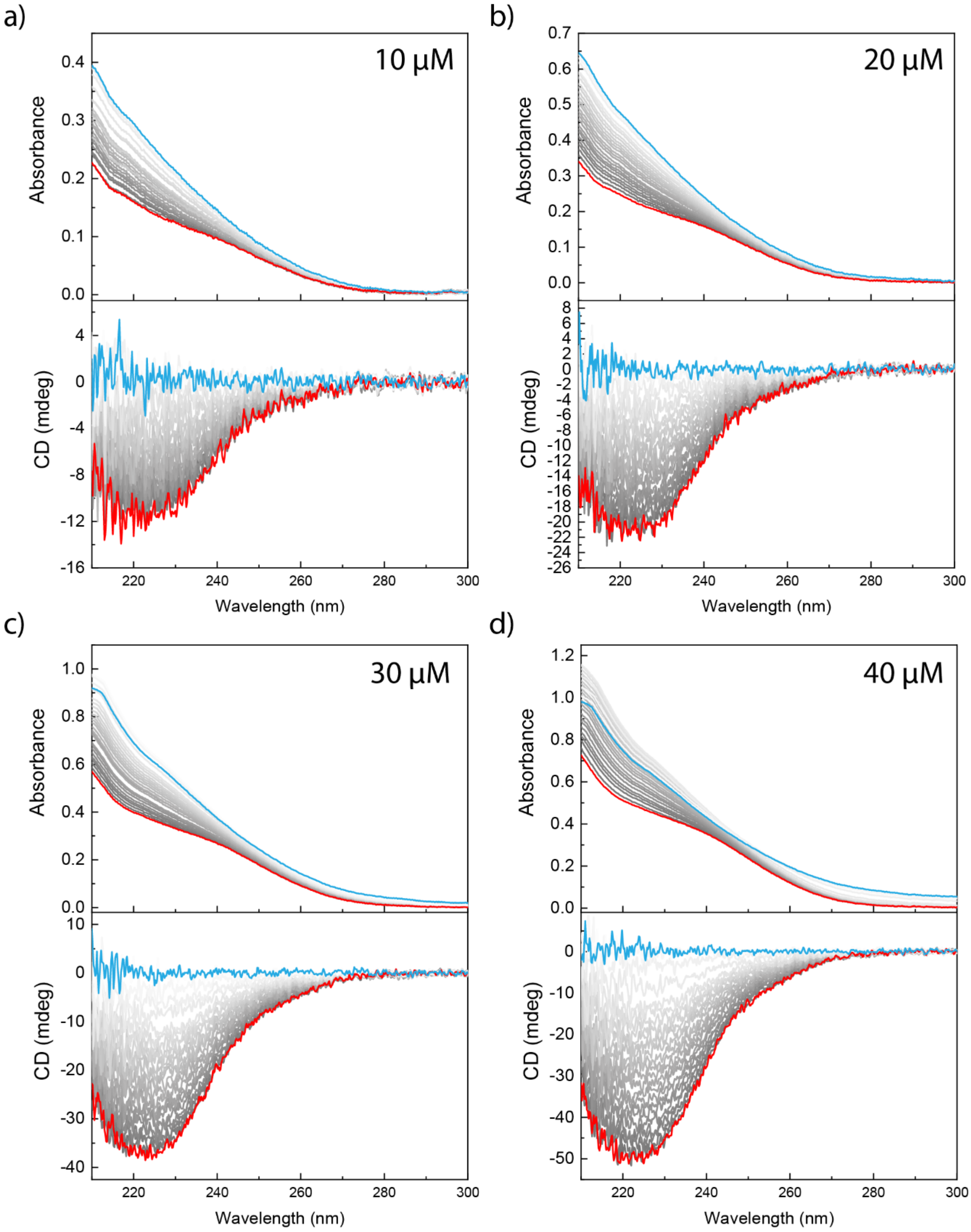

Figure S14: UV (top panels) and CD (bottom panels) spectra of the samples in the titration of BTA with Hex_4NCl at 10 (a), 20 (b), 30 (c) and 40 (d) µM BTA. The red spectrum indicates the sample containing 0 equivalents Hex_4NCl and the blue spectrum indicates the spectrum with 3 equivalents of the ammonium salt, with the increasing grayscale indicating the increasing intermediate equivalents (see also **Figure S12**).

5.2. Kinetic Analysis of *in-situ* BTA Formation

The kinetic model of *in-situ* synthesis of BTA contains three consecutive covalent reactions to produce BTA and 3 equivalents of ammonium chloride. The behavior of the products of this reaction can be divided into three different supramolecular events: Supramolecular polymerization of BTA, precipitation of ammonium chloride and hetero-complexation of BTA and ammonium chloride. The hetero-complexation of BTA and ammonium chloride is included as the formation of 1:1 dimers that then precipitate into 1:1 coassembly.
The ordinary differential equations (ODEs) from these (supra)molecular events were solved with ode15s in Matlab. An analytically derived Jacobian and a vector with tolerances were supplied to the ODE solver and the option NonNegative was used. From the concentration of BTA monomers in polymerized species, the CD signal was calculated using an estimated molar ellipticity θ for BTA in polymers of $-1.1 \cdot 10^{6}$ mdeg·M^{-1}·cm^{-1}. The molar ellipticity for free BTA monomers and BTA monomers in complex with ammonium chloride were set at 0 mdeg·M^{-1}·cm^{-1}, so the calculated CD signal is given by:

$$\mathrm{CD}_{\mathrm{calc}} = \theta_{\mathrm{polymer}} \cdot c_{\mathrm{polymer}} + \theta_{\mathrm{monomer}} \cdot c_{\mathrm{monomer}} + \theta_{\mathrm{complex}} \cdot c_{\mathrm{complex}} = -1.1 \cdot 10^{6} \cdot c_{\mathrm{polymer}}, \tag{5}$$

with θ_i the molar ellipticity of species *i* and c_i the molar concentration of species *i*.

Covalent reaction to synthesize BTAs
The chlorides of the BTA precursor benzene-1,3,5-tricarbonyl trichloride (Cl_3-BTA) are substituted by amines (R-NH_2) of the chiral side chains to consecutively form mono-, di- and tri-substituted BTAs (Cl_2-BTA, Cl_1-BTA, M_1) with the reaction rate constant k_{cov}:

$$\frac{d[\mathrm{Cl_2\text{-}BTA}]}{dt} = k_{\mathrm{cov}}[\mathrm{R\text{-}NH_2}][\mathrm{Cl_3\text{-}BTA}], \tag{6}$$

$$\frac{d[\mathrm{Cl_1\text{-}BTA}]}{dt} = k_{\mathrm{cov}}[\mathrm{R\text{-}NH_2}][\mathrm{Cl_2\text{-}BTA}], \tag{7}$$

$$\frac{d[\mathrm{M_1}]}{dt} = k_{\mathrm{cov}}[\mathrm{R\text{-}NH_2}][\mathrm{Cl_1\text{-}BTA}]. \tag{8}$$

Each reaction step yields ammonium chloride (T) as a byproduct:

$$\frac{d[\mathrm{T}]}{dt} = \frac{d[\mathrm{Cl_2\text{-}BTA}]}{dt} + \frac{d[\mathrm{Cl_1\text{-}BTA}]}{dt} + \frac{d[\mathrm{M_1}]}{dt}. \tag{9}$$

As previously shown, the mono- and di-substituted BTAs do not participate in the self-assembly any of the supramolecular interactions.

Supramolecular polymerization of BTA
BTA aggregates via a nucleation-elongation mechanism, resulting in the formation of long, ordered fibers. The reported binding constant K_e for BTA in methylcyclohexane is $1.05 \cdot 10^{6}$ M^{-1} at room temperature.[5] We assume a nucleus size of three BTA monomers[6], with an overall cooperativity of $3.7 \cdot 10^{-6}$ from CD cooling curves and $3.0 \cdot 10^{-6}$ from UV/vis cooling curves.[7] For a nucleus size of three, the cooperativity is then approximately $1.6 \cdot 10^{-2}$ for each nucleation step.[2]
To describe the supramolecular polymerization in a finite number of ODEs, only aggregates up to a set length are described individually in ODEs. Aggregates longer than this maximum specified length are included as the number concentration and mass concentration of large fibrils, as reported by Korevaar and co-workers.[8] Following the reported approach, the time-dependency of the free monomer is described with:

$$\frac{d[\mathrm{M}_1]}{dt} = -k_\mathrm{n}[\mathrm{M}_1](2[\mathrm{M}_1] + \sum_{i=2}^{n-1}[\mathrm{M}_i]) + k_{-\mathrm{n}}(2[\mathrm{M}_2] + \sum_{i=3}^{n}[\mathrm{M}_i]) - k_\mathrm{e}[\mathrm{M}_1](\sum_{i=n}^{N}[\mathrm{M}_i] + [F]) + k_{-\mathrm{e}}(\sum_{i=n+1}^{N}[\mathrm{M}_i] + [F]), \tag{10}$$

and the pre-nucleus oligomers with:

$$\frac{d[\mathrm{M}_i]}{dt} = k_\mathrm{n}[\mathrm{M}_1]([\mathrm{M}_{i-1}] - [\mathrm{M}_i]) + k_{-\mathrm{n}}([\mathrm{M}_{i+1}] - [\mathrm{M}_i]). \tag{11}$$

The time-dependency of the concentration of the nucleus of size n is then:

$$\frac{d[\mathrm{M}_n]}{dt} = k_\mathrm{n}[\mathrm{M}_1][\mathrm{M}_{n-1}] - k_{-n}[\mathrm{M}_n] + k_{-\mathrm{e}}[\mathrm{M}_{n+1}] - k_\mathrm{e}[\mathrm{M}_1][\mathrm{M}_n]. \tag{12}$$

For polymers larger than the nucleus size n, the time-dependent concentration is:

$$\frac{d[\mathrm{M}_i]}{dt} = k_\mathrm{e}[\mathrm{M}_1]([\mathrm{M}_{i-1}] - [\mathrm{M}_i]) + k_{-\mathrm{e}}([\mathrm{M}_{i+1}] - [\mathrm{M}_i]), \tag{13}$$

and for the longest specified polymers of length N:

$$\frac{d[\mathrm{M}_N]}{dt} = k_\mathrm{e}[\mathrm{M}_1]([\mathrm{M}_{N-1}] - [\mathrm{M}_N]) + k_{-\mathrm{e}}((1-\alpha)[F] - [\mathrm{M}_N]). \tag{14}$$

The time-dependency for the fibril number concentration $[F]$ is:

$$\frac{d[F]}{dt} = k_\mathrm{e}[\mathrm{M}_1][\mathrm{M}_N] - k_{-\mathrm{e}}(1-\alpha)[F], \tag{15}$$

and for the fibril mass concentration $[Z]$:

$$\frac{d[Z]}{dt} = k_\mathrm{e}[\mathrm{M}_1]((N+1)[\mathrm{M}_N] + [F]) - k_{-\mathrm{e}}([F] + N(1-\alpha)[F]). \tag{16}$$

$[\mathrm{M}_i]$ is the concentration of aggregated species with length i, k_n and k_e the rate constants of nucleation and elongation respectively, $k_{-\mathrm{n}}$ and $k_{-\mathrm{e}}$ the rate constants of dissociation in the nucleation and elongation phase respectively and α is given by:

$$\alpha = 1 - \frac{[F]}{[Z] - N[F]}. \tag{17}$$

Precipitation of ammonium salt

Precipitation of crystalline material involves a highly cooperative nucleation-growth mechanism where supersaturation of a compound results in the formation of nuclei, which rapidly grow to form larger crystals and precipitate out of the solution.[9] Classical nucleation theory (CNT) offers a simplified description for the formation of precipitates. CNT assumes nucleation in a closed system that follows the Szilard model for nucleation.[10] In this model, coagulation and fragmentation are not included, and instead a chain reaction of monomer adsorption/emission is considered. The stepwise growth of dissolved aggregates occurs up to a critical nucleus size (n_prec), typically with a considerably small binding constant $K_\mathrm{precn} = k_\mathrm{precn}/k_{-\mathrm{precn}}$:

$$\mathrm{T} + \mathrm{T} \underset{k_{-\mathrm{precn}}}{\overset{k_\mathrm{precn}}{\rightleftharpoons}} \mathrm{T}_2 \underset{k_{-\mathrm{precn}}}{\overset{k_\mathrm{precn}}{\rightleftharpoons}} \ldots \underset{k_{-\mathrm{precn}}}{\overset{k_\mathrm{precn}}{\rightleftharpoons}} \mathrm{T}_{n_\mathrm{prec}}. \tag{18}$$

Once a critical nucleus $\mathrm{T}_{n_\mathrm{prec}}$ is formed, the nucleation rate J_prec describes the conversion of this aggregate into a supernucleus that precipitates. In the generally accepted expression of CNT, J_prec relates the nucleation rate to the supersaturation via:[10]

$$J_{\text{prec}} = A_1 S \exp\left(\frac{-A_2}{\ln^2(S)} \right), \tag{19}$$

where A_1 and A_2 are kinetic and thermodynamic parameters respectively and S is the supersaturation of the ammonium salt, which is defined as the ratio between the actual concentration $[\text{HNEt}_3\text{Cl}]_{\text{ac}}$ and the equilibrium concentration $[\text{HNEt}_3\text{Cl}]_{\text{eq}}$:

$$S = \frac{[\text{HNEt}_3\text{Cl}]_{\text{ac}}}{[\text{HNEt}_3\text{Cl}]_{\text{eq}}}. \tag{20}$$

The widely used expression for J_{prec} in Eq. (19) results from a series of substitutions and mathematical operations on the general expression for one-component nucleation, proposed in the pioneering work from Farkas on cluster growth in nucleation following the Szilard model:[9,11]

$$J_{\text{prec}} = k_{\text{prec}}[\text{T}_{n_{\text{prec}}}] - k_{-\text{prec}}[\text{T}_{n_{\text{prec}}+1}]. \tag{21}$$

In this expression, $[\text{T}_{n_{\text{prec}}}]$ is the concentration of aggregates of the critical nucleus size, $[\text{T}_{n_{\text{prec}}+1}]$ is the concentration of the smallest supernuclei and k_{prec} and $k_{-\text{prec}}$ are the rate constants for transformation of the nuclei into supernuclei and backwards, respectively. CNT assumes $k_{-\text{prec}} << k_{\text{prec}}$, simplifying Eq. (21) to:

$$J_{\text{prec}} = k_{\text{prec}}[\text{T}_{n_{\text{prec}}}]. \tag{22}$$

Conveniently, the ODE approach which we use for this model calculates the concentration for $\text{T}_{n_{\text{prec}}}$ at every time step. Since the approximate Eq. (22) requires only one parameter, k_{prec}, to include the nucleation rate in the model, this expression for the nucleation rate was used in the model.

The growth of supernuclei is described by Lasaga in 1998 as a reaction on a surface with reaction rate R_{prec}:[12]

$$R_{\text{prec}} = k_{\text{precg}} A_{\text{prec}}([\text{T}]_{\text{S}} - [\text{T}]_{\text{eq}}), \tag{23}$$

where k_{precg} is the surface reaction rate constant, A_{prec} the surface area of the crystal and $[\text{T}]_{\text{S}}$ and $[\text{T}]_{\text{eq}}$ are the concentration at the surface and in equilibrium respectively. Since we are not interested in the exact size of the precipitates, the A_{prec} is replaced by the concentration of ammonium salt monomers present in crystals $[\text{T}]_{\text{cr}}$:

$$R_{\text{prec}} = \frac{d[\text{T}]_{\text{prec}}}{dt} = -\frac{d[\text{T}]}{dt} = k_{\text{precg}}[\text{T}]_{\text{prec}}([\text{T}]_{\text{S}} - [\text{T}]_{\text{eq}}). \tag{24}$$

Precipitation of BTA/ammonium salt complexes

The precipitates that were present in the samples from 30 to 200 μM imply that a model of a hetero-dimerization is too simple, and that the hetero-interaction should be modeled as the formation of larger aggregates instead. To simplify the two-component aggregation, BTA and ammonium chloride are assumed to first form hetero-dimers that subsequently aggregate with to form larger aggregates. The hetero-dimerization is an equilibrium with association and dissociation rate constants k_{dim} and $k_{-\text{dim}}$ respectively:

$$\text{M}_1 + \text{T} \underset{k_{-\text{dim}}}{\overset{k_{\text{dim}}}{\rightleftharpoons}} \text{M}_1 \cdot \text{T}. \tag{25}$$

This interaction can be described with the following ordinary differential equation:

$$\frac{d[\mathrm{M}_1 \cdot \mathrm{T}]}{dt} = -\frac{d[\mathrm{M}_1]}{dt} = -\frac{d[\mathrm{T}]}{dt} = k_{\mathrm{dim}}[\mathrm{M}_1][\mathrm{T}] - k_{-\mathrm{dim}}[\mathrm{M}_1 \cdot \mathrm{T}]. \tag{26}$$

This approach maintains the 1:1 stoichiometry without considering parameters for each of the possible interactions. The successive aggregation steps towards the critical nucleus size (n_{het}) follow the same mechanism as the precipitation of the ammonium salt and CNT with binding constant $K_{\mathrm{hetn}} = k_{\mathrm{hetn}}/k_{-\mathrm{hetn}}$:

$$[\mathrm{M}_1 \cdot \mathrm{T}] + [\mathrm{M}_1 \cdot \mathrm{T}] \underset{k_{-\mathrm{hetn}}}{\overset{k_{\mathrm{hetn}}}{\rightleftharpoons}} [\mathrm{M}_1 \cdot \mathrm{T}]_2 \underset{k_{-\mathrm{hetn}}}{\overset{k_{\mathrm{hetn}}}{\rightleftharpoons}} \ldots \underset{k_{-\mathrm{hetn}}}{\overset{k_{\mathrm{hetn}}}{\rightleftharpoons}} [\mathrm{M}_1 \cdot \mathrm{T}]_{n_{\mathrm{het}}}. \tag{27}$$

The nucleation rate J_{het} for transformation of the nuclei to the supernuclei is:

$$J_{\mathrm{het}} = k_{\mathrm{het}}[\mathrm{M}_1 \cdot \mathrm{T}]_{n_{\mathrm{het}}}, \tag{28}$$

with $[\mathrm{M}_1\mathrm{T}]_{n_{\mathrm{het}}}$ is the concentration of aggregates of the critical nucleus size and k_{het} is the rate constant for transformation of the nuclei into supernuclei. The growth rate of the larger aggregates, R_{het}, is:

$$R_{\mathrm{het}} = k_{\mathrm{hetg}}[\mathrm{M}_1 \cdot \mathrm{T}]_{\mathrm{cr}}([\mathrm{M}_1 \cdot \mathrm{T}]_{\mathrm{S}} - [\mathrm{M}_1 \cdot \mathrm{T}]_{\mathrm{eq}}), \tag{29}$$

where k_{hetg} is the surface reaction rate constant, $[\mathrm{M}_1 \cdot \mathrm{T}]_{\mathrm{S}}$ and $[\mathrm{M}_1 \cdot \mathrm{T}]_{\mathrm{eq}}$ are the concentration of hetero-dimers at the surface and in equilibrium respectively and $[\mathrm{M}_1 \cdot \mathrm{T}]_{\mathrm{cr}}$ is the amount of hetero-dimers present in the larger aggregate.

Obtained optimal values of fitting parameters				
Parameter (unit)	Optimized value		Parameter (unit)	Optimized value
k_{cov} ($M^{-1}s^{-1}$)	$6.75 \cdot 10^{2}$		K_{dim} (M^{-1})	$1.20 \cdot 10^{5}$
k_{e} ($M^{-1}s^{-1}$)	$1.41 \cdot 10^{5}$		K_{hetn} (M^{-1})	$1.19 \cdot 10^{4}$
k_{dim} ($M^{-1}s^{-1}$)	$2.57 \cdot 10^{2}$		K_{precn} (M^{-1})	$1.67 \cdot 10^{3}$
k_{hetn} ($M^{-1}s^{-1}$)	$2.21 \cdot 10^{3}$		$[M_1T]_{\mathrm{eq}}$ (M)	$7.26 \cdot 10^{4}$
k_{precn} ($M^{-1}s^{-1}$)	$9.73 \cdot 10^{4}$		$[T]_{\mathrm{eq}}$ (M)	$2.11 \cdot 10^{-5}$
k_{het} (s^{-1})	$6.32 \cdot 10^{4}$			
k_{prec} (s^{-1})	$6.59 \cdot 10^{4}$			
k_{hetg} ($M^{-1}s^{-1}$)	$3.31 \cdot 10^{4}$			
k_{precg} ($M^{-1}s^{-1}$)	$2.87 \cdot 10^{4}$			

Table S1: Optimized parameter obtained in the fitting.

Speciation plots for *in-situ* synthesis of BTA

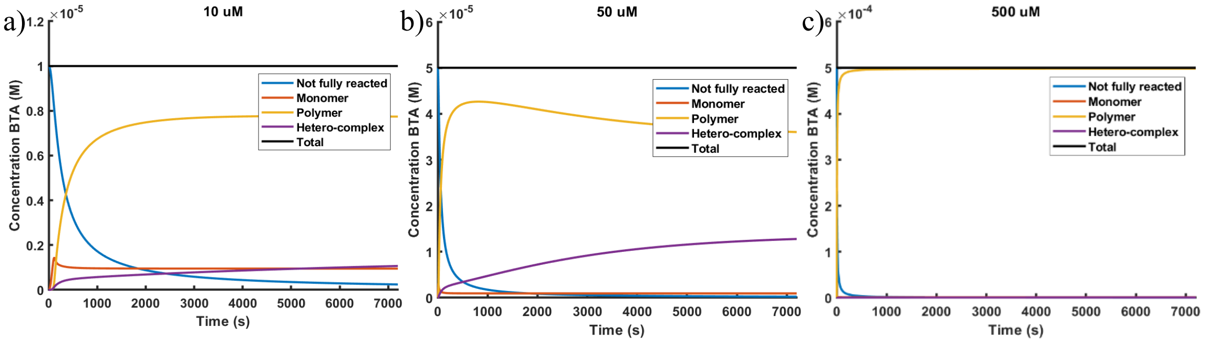

Figure S15: Speciation plots of BTA at a) 10, b) 50, and c) 500 μM, collected from simulations of kinetic model with the manually optimized parameter set. Species considered are not fully reacted BTA precursor (blue), free BTA monomer (red), self-assembled BTA monomer (yellow), BTA monomer in hetero-complex with ammonium chloride (purple) and all BTA species (black) in total.

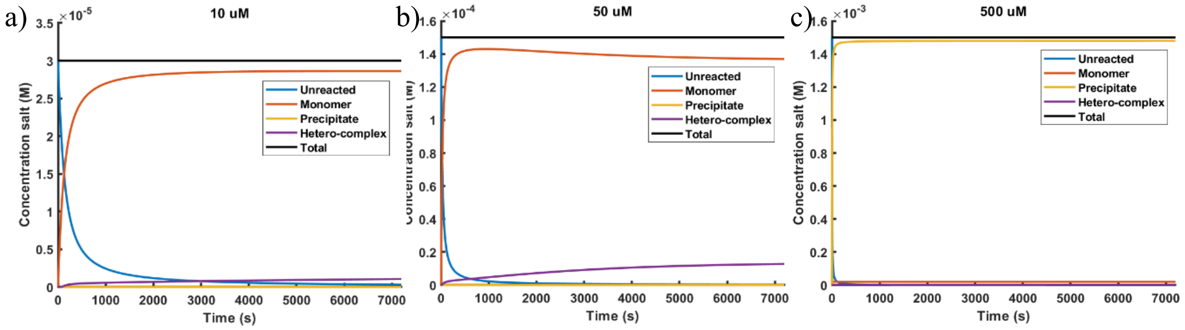

Figure S16: Speciation plots of $HNEt_3Cl$ in a) 10, 2) 50, and 3) 500 µM samples, collected from simulations of kinetic model with the manually optimized parameter set. Species considered are unreacted chiral amine (blue), free ammonium chloride (red), precipitated ammonium chloride (yellow), ammonium chloride in hetero-complex with BTA (purple) and all ammonium chloride species (black) in total.

Effect of parameter changes on the kinetic models

Although the models obtained with the initial sets of parameters reflect the trends observed in the experimental *in-situ* BTA formation, we decided to perform a systematic screening of the parameters to potentially further optimize the model. As the BTA polymerization and the precipitation of the ammonium salt have been studied before, we focused on the parameters for the formation of the BTA/$HNEt_3Cl$ hetero-complex. When plotting the simulated CD signal (after 1 h) against a series of kinetic parameters, we found that k_{hetn}, K_{hetn}, k_{het} and k_{hetg} have only minute influence on the simulated system (Figure S17). Significant changes in the simulated CD signal appeared for k_{dim}, K_{dim} and $[M_1T]_{eq}$ (Figure S18). Interestingly, the plot for the rate constants of the hetero-dimerization (k_{dim}) showed an unexpected step-shaped profile. We found that the k_{dim} needs to be above a certain threshold (approx. 198 1/s) to compete with the precipitation of $HNEt_3Cl$. Thus, k_{dim} is a decisive factor for the hetero-complexation to occur. Secondly, K_{dim} positively correlates with the hetero-complexation, which indicates that stronger binding of $HNEt_3Cl$ to BTA leads to a larger amount of BTA in hetero-complexes. Lastly, the equilibrium concentration of hetero-dimers ($[M_1T]_{eq}$) shows a negative correlation with the hetero-complexation. A higher equilibrium concentration reduces the tendency of the hetero-dimers to precipitate. In conclusion, k_{dim} is the deciding factor if hetero-precipitation occurs, while K_{dim} and $[M_1T]_{eq}$ strongly affect the number of molecules that participate in the hetero-precipitation.

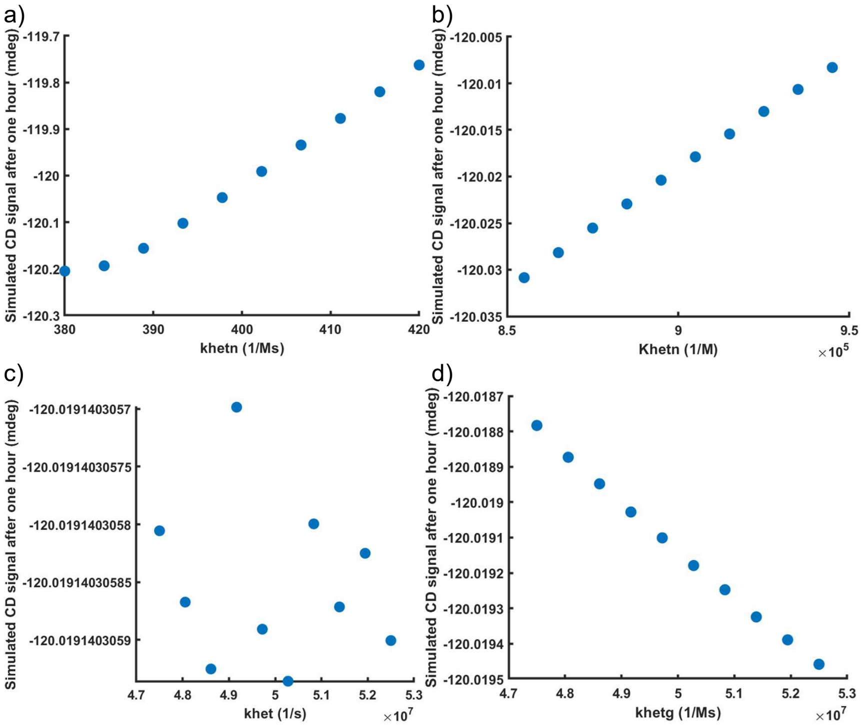

Figure S17: Screening of the sensitivity of the simulated CD signal to changes in the hetero-complexation parameters. The parameters were varied between 95 and 105% of the optimized values (Table S1). The graphs display the simulated CD signal at t = 3600 seconds. The analyzed parameters are a) rate constant for nucleation of larger hetero-complexes (k_{hetn}), b) binding constant of hetero-complex nuclei (K_{hetn}), c) rate constant for growth of larger hetero-complexes (k_{het}) and d) rate constant for addition of hetero-dimer to large hetero-complexes (k_{hetg}).

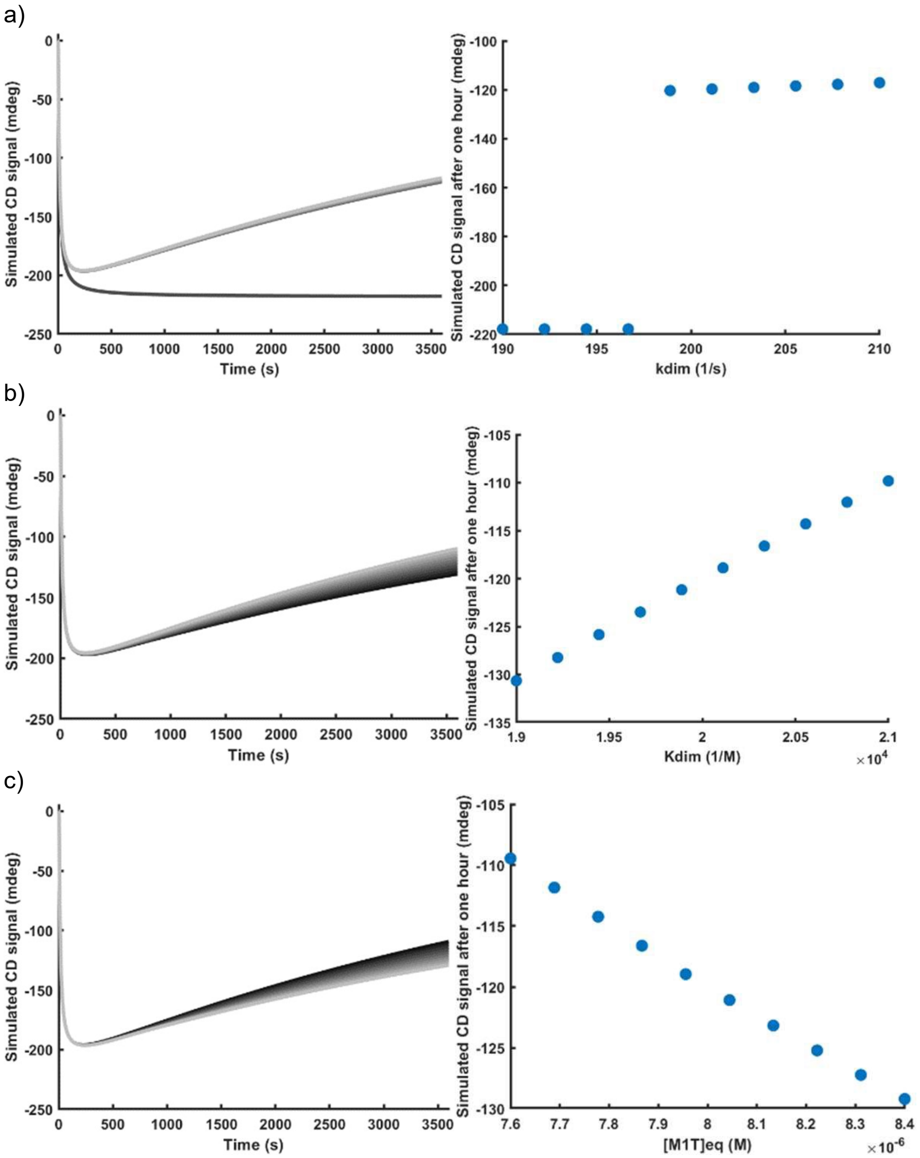

Figure S18: Screening of the sensitivity of the simulated CD signal to changes in the hetero-complexation parameters. The parameters were varied between 95 and 105% of the optimized values (Table S1). The left column displays the simulated CD signals from t = 0 to t = 3600 seconds and the right column displays the simulated CD signal at t = 3600 seconds. The analyzed parameters are a) rate constant for hetero-dimerization (k_{dim}), b) binding constant of hetero-dimers (K_{dim}) and c) equilibrium concentration of hetero-dimers ($[M1T]_{eq}$).

Separate fitting of the hetero-complex formation

The large number of fitting parameters used in the model (page S19–S22) potentially yields non-converged solutions. Thus, we performed additional examinations of the model. We investigated if the model can reliably find the global minimum by only fitting the parameters for hetero-complexation, which were established as the most critical parameters in the previous section. The parameters for the BTA formation, BTA assembly and $HNEt_3Cl$ precipitation were set at the values obtained in the manual fit (Table S1). In this fitting procedure, we prioritized the data points of the first 1000 seconds to increase the chance on mimicking the characteristic feature observed at intermediate concentrations. Similar optimized traces were found when the fitting procedure was initiated from different starting points, suggesting that the model indeed converges (Figure S19). The simulated CD signals at intermediate concentration show a strong depletion of the CD signal, which is even more drastic than the originally observed depletion. This feature is also present in the experimental curves (Figure 3), but in that case the signal remains constant at a concentration-dependent value (≠ 0) while the signal in the

model completely disappears. This clear difference between the experimental and simulated curves indicates that the model is oversimplified. The experimental curves imply that a compound is being consumed during the precipitation of the hetero-complexes, leading to the sharp transitions that are absent in both, the simulated and experimental curves. Although it is inconclusive what exactly causes this behavior, we suppose that the water content of the solutions could play a role, as this affects the solubility of the different precipitates (compare also ref 30).

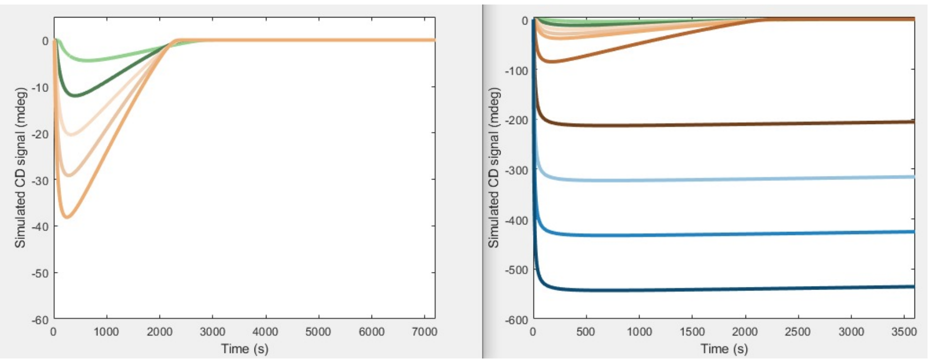

Figure S19: Simulated CD signal at λ = 223 nm at a concentration of 10 to 500 μM for the BTA precursor (light green to dark blue) in methylcyclohexane. Simulations were performed with Model 2 using optimized parameters for the hetero-complexation.

Water as a stimulus to direct the composition of the system

In the kinetic experiments, standard methylcyclohexane (with a water content of approximately 35 ppm) was used as the solvent in the sample preparation. Remarkably, majorly different results were obtained when 'dry' MCH (low water content of approximately 8 ppm) or wet MCH (high water content of approximately 59 ppm) were used as the solvent (Figure S20). The water contents were determined with a Karl Fisher-titrator. Counterintuitively, the effect that we ascribed to the hetero-aggregation is significantly reduced in samples of 200 μM in wet MCH, while the effect is completely absent in samples with dry MCH. The water content seems to affect the different rate constants involved in the system, which makes it a potential stimulus to alter the product composition.

To investigate if our kinetic model also can cover the effect of water on the in-situ BTA synthesis, we performed the following analysis:

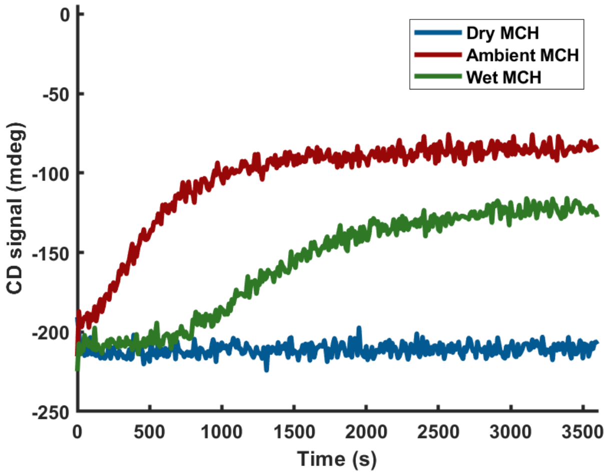

Figure S20: CD signal at λ = 223 nm followed over time at a concentration of 200 μM for the BTA precursor in dry (blue), standard (red) and wet (green) methylcyclohexane.

The impact of a solvent on a rate constant *k* is expressed in the *m*-value. The *m*-value, together with the volume fraction *f* of the corresponding co-solvent and the reaction rate in the pure main solvent k^0, are used to calculate the reaction rate in the resulting co-solvent for association and dissociation respectively:[13]

$$\log(k_a) = \log(k_a^0) - m_a f, \tag{30}$$

$$\log(k_d) = \log(k_d^0) + m_d f. \tag{31}$$

Eqs. (30) and (31) were used in the kinetic model to calculate k_n, k_{-n}, k_e, k_{-e}, k_{dim}, k_{-dim}, k_{hetn}, k_{-hetn}, k_{het}, k_{hetg}, k_{precn}, k_{-precn}, k_{prec} and k_{precg}. By manually adjusting the *m*-values for the competing interactions, we found that a similar trend was obtained as the experimental results with *m*-values of 0 kJ/mol for the supramolecular polymerization of BTA, 10 kJ/mol for association and dissociation of hetero-complexes of BTA and ammonium chloride, 80 kJ/mol for ammonium chloride aggregation and 20 kJ/mol for ammonium chloride aggregate dissociation (Figure S21). For samples in dry MCH, the self-assembly of BTA is observed without a reduction of the CD signal. For samples in ambient MCH, the same curve is obtained as previously observed with the experiments where the concentration was varied. For samples in wet MCH, a similar curve is observed where the reduction of the CD signal is significantly lower than the sample containing ambient MCH.

The speciation plots of BTA and ammonium chloride in these simulations shows the composition of the samples during the time interval of the experiments (Figure S22). For the samples in dry MCH, precipitation of ammonium chloride is observed with minimal formation of hetero-complexes. Simulations of the samples in ambient MCH show self-assembly of fast BTA with gradual formation of hetero-complexes. Ammonium chloride does not precipitate in the samples in ambient MCH. For the samples in wet MCH, similar speciation curves are observed as in ambient MCH with a reduced formation of hetero-complexes.

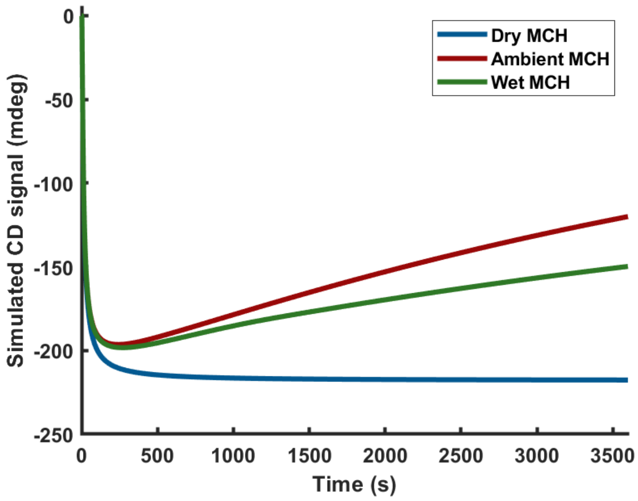

Figure S21: Simulated CD signal at λ = 223 nm at a concentration of 200 μM for the BTA precursor in dry (blue, 8 ppm water), standard (red, 39 ppm water) and wet (green, 59 ppm water) methylcyclohexane. Simulations were performed with Model 2 using m-values of 0 kJ/mol for the supramolecular polymerization, 10 kJ/mol for both association and dissociation of hetero-complexes, 80 and 20 kJ/mol for association and dissociation of ammonium chloride aggregates respectively.

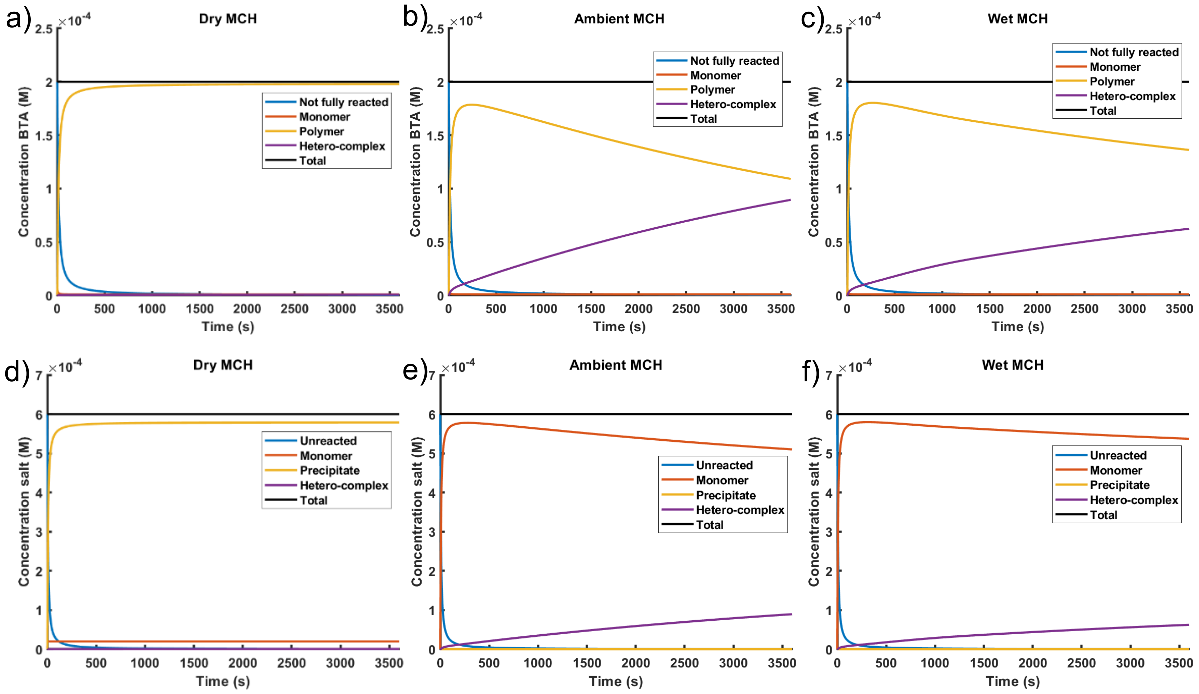

Figure S22: Speciation plots of a,b,c) BTA and d,e,f) ammonium chloride for 200 µM samples in a,d) dry, b,e) ambient and c,f) wet methylcyclohexane. The speciation was collected from simulations of Model 2 with additional inclusion of the effect of water on the rate constants.

The speciation plots give valuable insights into the effect of water on the composition of the supramolecular system. The speciation of ammonium chloride seems to indicate that low amounts of water in MCH increase the solubility of ammonium chloride in the solvent enough to prevent precipitation of the salt. When the water content of MCH is very low, however, the solubility of the salt decreases and precipitation of ammonium chloride occurs. Consequently, ammonium chloride does not interact with BTA to form hetero-complexes and BTA is fully able to participate in self-assembly. The water content of MCH in ambient conditions solubilizes ammonium chloride, facilitating the formation of hetero-complexes of ammonium chloride and BTA. When the water content of MCH is higher than in ambient conditions, however, the hetero-complexation of ammonium chloride and BTA is reduced, which could imply that water destabilizes the hetero-complexes or that water stabilizes the supramolecular polymers of BTA. As a result, the competition between these pathways favors BTA self-assembly. Carefully considering the water content of the solvent appears critical in supramolecular systems with competing pathways and the results suggest that the water content can be used as a stimulus to control the composition of the supramolecular system.

6. DFT Computational Analysis

Computational analysis of BTA monomer, $HNEt_3Cl$, BTA dimer and BTA/$HNEt_3Cl$ adduct was performed with Schrödinger Maestro Suite 2021-2. The input structures were generated using the built-in 2D structure generator followed by geometry minimization with Macromodel (OPLS4 force field, vacuum). The BTA dimer structure was oriented such that linear hydrogen bonds along the helical screw axes are formed. Optimization of all structures was performed using Jaguar at the B3-LYP-D3 /6-311G+* level of theory. Frequency calculations on the optimized structures yielded no imaginary frequencies, indicating stationary points on the potential energy surface. All total free energy values are shown in kcal/mol. The binding energy of the dimer and the BTA/$HNEt_3Cl$ adduct was calculated by subtraction of the energies of the corresponding components from that of the dimer. The energy gain for the BTA/$HNEt_3Cl$ complex is about 40% of that of the BTA dimerization. This is in good agreement with the titration experiments (compare manuscript Figure 4), which shows that between 2.0 and 3.0 equiv. of $HNEt_3Cl$ are required to fully disassemble the BTA-based supramolecular polymer.

Note that the calculated values differ from the mass balance models (compare 4.) as they are based on BTA model compounds (methyl versus C_{10}-chain) and calculated in gas phase.

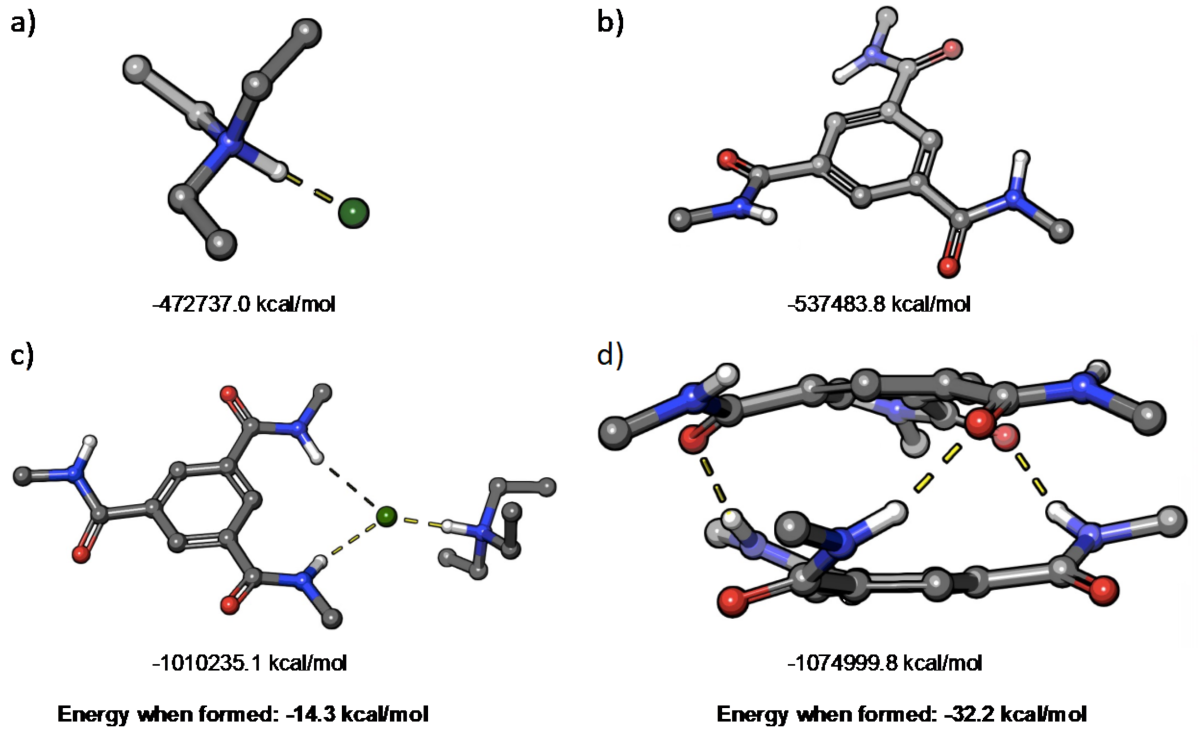

Figure S23: Lowest energy structures obtained by DFT-based geometry optimization (B3-LYP-D3 /6-311G+*). The energies of $HNEt_3Cl$ (a), BTA (b), BTA/$HNEt_3Cl$ complex (c), and BTA dimer (d) are given in kcal/mol. The energy gain for the formation of the di-molecular complexes c) and d) are obtained by substratcting the energies of the correponding monomers from the dimer energies (for c: E(BTA/$HNEt_3Cl$ complex) – [E(BTA) + E($HNEt_3Cl$)], for d: E(BTA dimer) – [2 * E(BTA)]).

xyz coordinates of $HNEt_3Cl$:

C	-0.48660	0.76960	-0.53500
C	0.40050	0.27390	0.59860
N	1.82900	0.02210	0.22100
C	2.57950	1.29020	-0.05950
C	4.09080	1.09540	-0.12000
C	2.02190	-1.01930	-0.83990
C	1.30880	-2.32890	-0.52790
H	2.27800	-0.40630	1.14010
Cl	2.96200	-1.14820	2.61700
H	-1.49840	0.92340	-0.15300
H	-0.14630	1.72250	-0.94600
H	-0.55420	0.04850	-1.35250
H	0.43240	0.98930	1.42190
H	0.02570	-0.65650	1.02330
H	2.19330	1.72300	-0.98600
H	2.33110	1.96750	0.75950
H	4.57030	2.07710	-0.12680
H	4.44460	0.54660	0.75490
H	4.41150	0.57150	-1.02230
H	1.70970	-0.59700	-1.79920
H	3.09470	-1.20000	-0.88490
H	1.63810	-3.08780	-1.24130
H	1.56490	-2.67240	0.47660
H	0.22310	-2.24940	-0.61260

xyz coordinates of BTA:

C	-0.49200	-1.06640	0.51310
C	0.23190	0.12120	0.60100
C	-0.45680	1.33560	0.68810
C	-1.84980	1.36920	0.65650
C	-2.55990	0.16720	0.57370
C	-1.89020	-1.05300	0.51180
C	-2.66150	2.63670	0.73840
N	-2.01860	3.80040	0.44660
O	-3.84410	2.61180	1.05920
C	-2.72700	5.07110	0.47110
C	-2.58830	-2.38500	0.41980
N	-3.87890	-2.43080	0.85010
O	-2.01460	-3.37290	-0.02370
C	1.73780	0.18520	0.63970
N	2.41250	-0.91150	0.19780
O	2.32020	1.18220	1.05020
C	3.86720	-0.92730	0.16400
C	-4.62720	-3.67860	0.83320
H	-0.00720	-2.03480	0.47210
H	0.13810	2.23280	0.81470
H	-3.64130	0.23000	0.54350
H	-1.10030	3.77090	0.03520
H	-2.00290	5.88150	0.38570
H	-3.45060	5.14490	-0.34620
H	-3.26910	5.17310	1.41140
H	-4.25870	-1.65020	1.36050
H	1.91430	-1.64380	-0.28080
H	4.26010	-0.23580	-0.58730
H	4.26300	-0.62960	1.13530
H	4.20430	-1.93820	-0.06550
H	-5.67320	-3.46660	1.05490
H	-4.55600	-4.13850	-0.15270
H	-4.23920	-4.39110	1.56730

xyz coordinates of $HNEt_3Cl$/BTA complex:

C	5.12200	-2.77460	-0.59970
C	5.53380	-2.37020	-2.00880
N	4.53560	-2.77770	-3.05760
C	4.19530	-4.23900	-3.07170
C	5.34920	-5.15170	-3.45270
C	4.89290	-2.19870	-4.39890
C	3.82170	-2.43000	-5.45610
H	3.62090	-2.28820	-2.77780
Cl	2.06550	-1.41580	-2.30520
H	5.78630	-2.28390	0.11490
H	4.10120	-2.45020	-0.38750
H	5.19860	-3.85030	-0.42980
H	5.59210	-1.28270	-2.07650
H	6.50860	-2.77550	-2.28820
H	3.81020	-4.47090	-2.07980
H	3.35360	-4.34840	-3.75400
H	5.00370	-6.18740	-3.43250
H	5.71990	-4.95170	-4.46040
H	6.18630	-5.07410	-2.75560
H	5.01330	-1.12820	-4.22520
H	5.86350	-2.60000	-4.69760
H	4.05770	-1.82490	-6.33410
H	3.76880	-3.47030	-5.78260
H	2.84140	-2.12090	-5.08720
C	-1.14130	0.49220	-1.16780
C	-2.02020	-0.18080	-0.31740
C	-2.89010	0.55890	0.48780
C	-2.87460	1.95460	0.45930
C	-1.96920	2.61370	-0.37570
C	-1.10210	1.88930	-1.19460
C	-3.78340	2.79920	1.31320
N	-4.98110	2.23980	1.65930
O	-3.46570	3.92320	1.67790
C	-5.95720	2.99300	2.43110
C	-0.17730	2.66320	-2.09880
N	0.89280	1.99190	-2.60480
O	-0.38670	3.84180	-2.36600
C	-2.10160	-1.68220	-0.23240
N	-1.06650	-2.38940	-0.75610
O	-3.06630	-2.23720	0.28860
C	-1.07510	-3.84020	-0.71780
C	1.82680	2.66410	-3.48880
H	-0.50590	-0.07310	-1.83900
H	-3.55390	0.00980	1.14560
H	-1.94400	3.69610	-0.39540
H	-5.28610	1.40480	1.18550
H	-6.38680	3.81630	1.85130
H	-6.75570	2.32090	2.74730
H	-5.47940	3.41950	3.31340
H	1.12450	1.05580	-2.30290
H	-0.22810	-1.93410	-1.08980
H	-1.03880	-4.21300	0.31040
H	-1.98480	-4.22950	-1.17970
H	-0.20590	-4.20370	-1.26610
H	1.29770	3.11350	-4.33170
H	2.36490	3.46360	-2.97030
H	2.54010	1.92750	-3.85930

xyz coordinates of BTA dimer:

C	-0.11110	0.07070	2.88570
C	-0.82890	1.26480	2.82320
C	-2.22540	1.22500	2.79300
C	-2.90010	0.00460	2.78050
C	-2.16870	-1.18430	2.84310
C	-0.77480	-1.15900	2.87310
C	-4.38780	-0.11460	2.58600
N	-4.99710	0.92990	1.96690
O	-5.00270	-1.12470	2.92300
C	-6.41940	0.88230	1.67830
C	0.07550	-2.39720	2.77640
N	-0.50870	-3.48140	2.20270
O	1.24720	-2.40520	3.14880
C	-0.17640	2.61230	2.66880
N	1.07270	2.61540	2.13420
O	-0.76500	3.65100	2.96280
C	1.76120	3.86980	1.88780
C	0.24920	-4.70430	2.00630
H	0.97130	0.06050	2.91840
H	-2.75730	2.16760	2.75340
H	-2.71910	-2.11700	2.84070
H	-4.44940	1.64260	1.49700
H	-6.66860	0.06280	0.99430
H	-6.99080	0.72590	2.59470
H	-6.71460	1.82850	1.22590
H	-1.38510	-3.39010	1.70030
H	1.44610	1.78340	1.69040
H	1.23120	4.48800	1.15380
H	1.83730	4.45110	2.80800
H	2.76140	3.65070	1.51520
H	1.10360	-4.55010	1.33730
H	0.64210	-5.06780	2.95720
H	-0.41080	-5.45740	1.57670
C	-0.93180	-1.34200	-0.51060
C	-0.01690	-0.29290	-0.49220
C	-0.49100	1.02100	-0.54310
C	-1.85650	1.28920	-0.58440
C	-2.75680	0.22010	-0.60190
C	-2.30740	-1.09670	-0.55060
C	-2.43010	2.65620	-0.31100
N	-1.61760	3.72340	-0.45620
O	-3.59120	2.77080	0.09160
C	-1.97690	5.03470	0.07550
C	-3.21430	-2.25920	-0.23580
N	-4.53960	-2.09760	-0.42900
O	-2.74640	-3.29760	0.24050
C	1.43190	-0.47930	-0.12070
N	1.95960	-1.71550	-0.23500
O	2.08210	0.46800	0.33010
C	3.23630	-2.06070	0.38310
C	-5.51240	-3.03240	0.12830
H	-0.62310	-2.36970	-0.37130
H	0.24110	1.80920	-0.42630
H	-3.80830	0.46640	-0.53380
H	-0.66370	3.57810	-0.74250
H	-1.34190	5.78910	-0.38930
H	-3.01830	5.24750	-0.16010
H	-1.83770	5.05390	1.15960
H	-4.88050	-1.21570	-0.77400
H	1.37890	-2.46780	-0.56620
H	3.60300	-2.98690	-0.05960
H	3.95540	-1.26470	0.19710
H	3.11090	-2.19240	1.46110
H	-6.46690	-2.89200	-0.37920
H	-5.16680	-4.05210	-0.03290
H	-5.63580	-2.85530	1.20000

7. References

[1] L. Brunsveld, A. P. H. J. Schenning, M. A. C. Broeren, H. M. Janssen, J. A. J. M.

Vekemans, E. W. Meijer, *Chem. Lett.* **2000**, *29*, 292.
[2] D. Zhao, J. S. Moore, *Org. Biomol. Chem.* **2003**, *1*, 3471.
[3] H. M. M. Ten Eikelder, B. Adelizzi, A. R. A. Palmans, A. J. Markvoort, *J. Phys. Chem. B* **2019**, *123*, 6627.
[4] K. V. Rao, M. F. J. Mabesoone, D. Miyajima, A. Nihonyanagi, E. W. Meijer, T. Aida, *J. Am. Chem. Soc.* **2020**, *142*, 598.
[5] L. N. J. De Windt, C. Kulkarni, H. M. M. Ten Eikelder, A. J. Markvoort, E. W. Meijer, A. R. A. Palmans, *Macromolecules* **2019**, *52*, 7430.
[6] K. K. Bejagam, S. Balasubramanian, *J. Phys. Chem. B* **2015**, *119*, 5738.
[7] C. Kulkarni, E. W. Meijer, A. R. A. A. Palmans, *Acc. Chem. Res.* **2017**, *50*, 1928.
[8] P. A. Korevaar, S. J. George, A. J. Markvoort, M. M. J. Smulders, P. A. J. Hilbers, A. P. H. J. Schenning, T. F. A. De Greef, E. W. Meijer, *Nature* **2012**, *481*, 492.
[9] D. Kashchiev, G. M. Van Rosmalen, *Cryst. Res. Technol.* **2003**, *38*, 555.
[10] D. Kashchiev, *Nucleation – Basic Theory with Applications*, Butterworth-Heinemann, **2000**.
[11] L. Farkas, *Zeitschrift für Phys. Chemie* **1927**, *125U*, 236.
[12] A. Lasaga, *Kinetic Theory in the Earth Sciences*, Princeton University Press, Princeton, New Jersey, **1998**.
[13] P. A. Korevaar, C. Schaefer, T. F. A. De Greef, E. W. Meijer, *J. Am. Chem. Soc* **2012**, *134*, 13482.
